# Modified Zhujing pill regulates RPE cholesterol metabolism and gut microbiota in an age-related macular degeneration mouse model

**DOI:** 10.3389/fcimb.2025.1691360

**Published:** 2025-10-31

**Authors:** Shuibin Cen, Shiqin Xie, Khalid S. Ibrahim, Michal R. Baran, Xing Li, James Reilly, Zhoujin Tan, Zhiming He, Xinhua Shu

**Affiliations:** ^1^ School of Materials, Guangdong Industry Polytechnic University, Guangzhou, Guangdong, China; ^2^ School of Traditional Chinese Medicine, Hunan University of Chinese Medicine, Changsha, Hunan, China; ^3^ Department of Biology, Faculty of Science, University of Zakho, Zakho, Iraq; ^4^ Department of Biological and Biomedical Sciences, Glasgow Caledonian University, Glasgow, United Kingdom; ^5^ Pu Ai Medical School, Shaoyang University, Shaoyang, Hunan, China

**Keywords:** age related macular degeneration, cholesterol, oxidative stress, inflammation, gut microbiota, modified Zhujing pill

## Abstract

**Background:**

Age-related macular degeneration (AMD) is a common retinal disorder, causing blindness in aged individuals. One of the traditional Chinese medicines, modified Zhujing pill (MZP), has been widely used to treat various ocular disorders, including AMD; however, its protective mechanisms remain elusive. In this study, we explored the functional role of MZP in high-fat-diet-fed mice, a commonly used model for AMD.

**Methods:**

Compounds of MZP water extract were identified by high-performance liquid chromatography (HPLC)/mass spectrometry (MS)/MS. The mice were divided into three groups: group 1 mice fed with control diet (CD), group 2 mice fed with high-fat diet (HFD), and group 3 mice fed with HFD for 12 weeks; groups 1 and 2 were then treated with physiological saline, while group 3 was treated with MZP for 4 weeks. The cholesterol level and expression of cholesterol homeostasis-associated genes, antioxidant genes, and proinflammatory cytokines in mouse tissues were measured using biochemical approaches. Mouse cecum microbiota compositions and metabolic functions were analyzed using 16rRNA sequencing and bioinformatics approach.

**Results:**

HFD-fed mice had high levels of cholesterol in the retinal pigment epithelial (RPE) cells, liver, and serum, a decreased expression of cholesterol homeostasis-associated genes and antioxidant genes in the RPE and liver, and an increased expression of proinflammatory cytokines. MZP treatment counteracted HFD-induced pathologic effects. Additionally, HFD altered cecum bacterial compositions and diversities associated with individual metabolic pathways. These metabolic pathways are involved in the biosynthesis of bacterial metabolites, mitochondrial function, oxidative stress, and inflammation. MZP reversed most of the changes back to control characteristics.

**Conclusion:**

We postulate that the beneficial effects of MZP against AMD are possibly related to lowering the cholesterol level, suppressing oxidative stress and inflammation, and modulating gut microbiota and associated functions.

## 1 Introduction

Age-related macular degeneration (AMD) is a severe retinal disorder, affecting over 200 million patients over 50 years old globally (Wong et al., 2014). The major clinical feature of AMD is abnormal focal and diffuse extracellular deposits underneath the retinal pigment epithelial (RPE) layer, which are thought to cause dysfunction and later atrophy of RPE, followed by loss of photoreceptors (Jiang et al., 2025). The retinal pathology of AMD patients demonstrates that cholesterol is enriched in these deposits, suggesting that dysregulation of cholesterol homeostasis may contribute to AMD pathogenesis (Curcio et al., 2005; Pang et al., 2015). Genome-wide association studies have demonstrated that some cholesterol metabolism/transport genes are associated with AMD (Fritsche et al., 2016). Cholesterol-lowering medications, such as statin treatment, confer a reduced risk in developing AMD (McGwin et al., 2005); conversely, a cholesterol-enriched diet is thought to increase the risk of developing AMD (Cao et al., 2022b). A high-cholesterol diet can also induce AMD-like features in rabbits (Dasari et al., 2011). This data further supports the hypothesis that cholesterol is involved in the formation and progression of AMD.

The RPE layer is a polarized single cell layer that predominantly functions in providing nutrients for photoreceptors and renewing photoreceptor outer segments. The RPE also plays a critical role in retinal cholesterol homeostasis. The RPE can perform *de novo* cholesterol synthesis as cholesterol synthesis pathway genes, such as HMGCR, DHCR24, and SQLE, are expressed in human and mouse RPE (Zheng et al., 2012, 2015; Louer et al., 2020); similarly, genes involved in cholesterol metabolism and trafficking—for example, *CYP27A1*, *CYP46A1*, *NR1H3* (encoding LXRα), *ABCA1*, and *ABCG1*—are also expressed in human and mouse RPE (Zheng et al., 2012; Biswas et al., 2017, 2021). RPE-specific knockout of mouse *Abca1* caused impaired cholesterol efflux and accumulation of cholesterol-ester-enriched lipid droplets in the RPE (Storti et al., 2019). The translocator protein (TSPO), a mitochondrial outer membrane protein, mediates cholesterol metabolism and trafficking in the RPE. Loss of TSPO in human RPE (ARPE-19) cells results in defective cholesterol efflux and an increased intracellular level of cholesterol (Biswas et al., 2017). Global knockout of *Tspo* in mice causes an increase in the levels of cholesterol, triglyceride, and phospholipids, downregulates the expression of cholesterol metabolism and trafficking genes, and increases inflammation in the RPE (Farhan et al., 2021). The RPE takes up LDL-bound cholesterol mainly from choroidal capillary vessels via LDLR-mediated endocytosis. Excess cholesterol in the RPE is returned to the liver via cholesterol reverse transport for storage as cholesterol esters or for excretion in the bile (Zhang et al., 2021).

As a complex retinal disorder, AMD is associated with complicated pathological mechanisms. It is putative that oxidative stress, inflammation, dysregulation of lipid metabolism, and angiogenesis are major contributors to the pathogenesis of AMD (Jiang et al., 2025). Consolidated data also suggests that gut dysbiosis is associated with AMD (Xiao et al., 2023). A comparison of gut microbiota between AMD patients and healthy controls has shown changes in microbiota composition—for example, Zhang et al. demonstrate that fecal samples of AMD patients have high levels of *Bacteroidota* and *Proteobacteria* and a low level of *Firmicutes* compared to that of the controls (Zhang et al., 2023). Evidence from AMD rodent models is consistent with the findings of clinical studies. High-fat-diet (HFD) -induced gut dysbiosis in mice exacerbates the formation of choroid neovascularization and microglia activation, both of which are key features of AMD (Andriessen et al., 2016). Studies from germ-free (GF) and specific pathogen-free (SPF) models provide direct evidence of gut microbiota associated with AMD. GF mice display reduced lesion size and microglia activation in laser-induced choroidal neovascularization compared to that of SPF mice; gut microbiota possibly is involved in the regulation of gene expression in the RPE, as over 660 genes are differentially expressed between GF and SPF mice in the RPE. Most of these genes function in inflammatory response, immune regulation, and angiogenesis (Zhang et al., 2022).

Current treatments for AMD include anti-VEGF (vascular endothelial growth factor) therapy (for neovascular AMD) and anti-complement therapy (for nonexudative AMD) via intravitreal injections (Boopathiraj et al., 2024). These treatments can cause adverse events and complications—for example, intraocular inflammation, retinal detachment, ocular hemorrhage, and cataract. Therefore, alternative effective treatments for AMD are required. Traditional Chinese medicine (TCM) has been used in the treatment of AMD patients for thousands of years in China, and 101 formulas have been reported, containing 167 Chinese medicines (Li et al., 2022). However, TCM has not been accepted for the treatment of AMD in Western countries, perhaps due to unelucidated complicated mechanisms. Zhujing pill (ZP) is one of the TCM formulas that is firstly described in the medicine book *Taiping Holy Prescriptions for Universal Relief*, edited by Huaiyin Wang in Song dynasty, and initially composed of three Chinese herbs: Tusizi (*Cuscuta chinensis* Lam), Dihuang (Radix Rehmanniae Praeparata), and Cheqianzi (*Plantago asiatical*) (Wang, 1958). Dr Da-Fu Chen, a famous ophthalmologist, modified the formula in his TCM ophthalmological book in 1978, removing one medicinal herb and adding seven others (Chen, 1978). Based on the theory of TCM, the therapeutic effects of ZP or modified ZP (MZP) on various disorders are through balancing Yin and Yang and tonifying the liver and kidney. ZP or MZP has shown protective effects against retinal disorders including retinitis pigmentosa (Fan and Zhang, 2018), diabetic retinopathy (Lei et al., 2018; Cui et al., 2023), and age-related macular degeneration (Feng et al., 2024). However, the underlying therapeutic mechanisms remain elusive.

In this study, we aimed to investigate the protective role of MZP against AMD in the regulation of cholesterol metabolism, suppression of both oxidative stress and inflammation, and modulation of gut microbiota. The HFD-fed mouse model, although utilizing young mice, recapitulates the major pathological features of AMD (Andriessen et al., 2016; Biswas et al., 2021; Sterling et al., 2022; Xiao et al., 2023). We therefore treated HFD-fed mice with MZP, measured the cholesterol level in the RPE, liver, and serum, quantified the expression of cholesterol-metabolism-associated genes in the RPE and liver, and examined the microbiota. We found that MZP modulated RPE cholesterol metabolism and gut microbiota. Our study can improve the understanding of the therapeutic effects of TCM and promote the recognition of TCM in treating AMD in Western countries.

## 2 Materials and methods

### 2.1 Preparation of MZP water extract

MZP, purchased from the First Affiliated Hospital of Hunan University of Chinese Medicine, included Chushizi (*Broussonetia papyrifera* (L.), Vent *Morus papyrifera* L., dried fruit) 15 g, Gouqizi (*Lycium chinense* Mill, dried fruit) 12 g, Tusizi (*Cuscuta chinensis* Lam, dried seeds) 15 g, Wuweizi (*Schisandra chinensis* (Turcz.) Baill, dried fruit) 12 g, Chongweizi (*Leonurus japonicus* Houtt, dried fruit) 10 g, Cheqianzi (*Plantago asiatical*, dried seeds) 12 g, Mugua (*Chaenomeles sinensis*, dried fruit) 12 g, Sanqi (*Panax notoginseng* (Burk.) F.H. Chen, dried roots) 3 g, and Hanshuishi (*Gypsum rubrum*) 10 g. MZP was initially soaked with 50 mL distilled water (dH_2_O) for 20 min, then boiled, and simmered for 30 min. The first batch of decoction was obtained by filtering through a cheesecloth. The herbal mixture was again soaked in 50 mL dH_2_O for 20 min and then boiled for 30 min at a low simmer. The second batch of decoction was strained. Both batches were mixed, concentrated to 1.10 g/mL, aliquoted, and stored in a freezer.

### 2.2 Compound identification in water-extracted MZP

Water extract of MZP (from Section 2.1) was concentrated and dried to form powder in a glass evaporating dish within a vacuum oven. Then, 2 mg of powder was initially dissolved in 1 mL ultrapure water and further diluted with acetonitrile to a final concentration of 2 µg/mL. The samples were run in duplicate and in both positive and negative ionization modes at a flow rate of 0.2 mL/min with a gradient elution scheme (**Supplementary Figure S1**) on a reverse-phase column (HALO 90 A RP-AMIDE 2 UM 2.1 X150M) and Thermo Scientific Q-exactive orbitrap mass spectrometer as the high-performance liquid chromatography/mass spectrometry (HPLC/MS)/MS setup. MZMINE 4.5.0 was used to process the data and identify the components with MASSBANK (https://massbank.eu/MassBank) and global natural products social molecular networking (GNPS) libraries (https://gnps.ucsd.edu) with the standard MZ Wizard settings for HPLC-Orbitrap-DDA.

### 2.3 Animal experiments

All animal work was approved by the Animal Welfare Committee of Hunan University of Chinese Medicine (license number: SYXK (Xiang) 2019-0009). C57BL/6 male mice (4 weeks old) were randomly divided into three groups (10 mice in each group), with one group (group 1) fed with chow diet and the other two groups (group 2 and group 3) fed with HFD containing 78.75% chow diet, 10% lard, 10% corn oil, 1% cholesterol, and 0.25% sodium cholate. After 12 weeks of feeding, groups 1 and 2 had daily intra-gastric administration of physiological saline for 4 weeks, while group 3 had daily intra-gastric administration of MZP water extract (14.69 g/kg body weight) for 4 weeks. The dose of MZP treatment was calculated based on the clinical dose in treating AMD patients using the formula: mouse dose (g/kg) = human dose (g/kg) × K_m_ ratio, guided by a previous publication (Nair and Jacob, 2016). During the treatment period, animals in group 1 were fed with a chow diet, and animals in group 2 and group 3 were fed with HFD. The body weight of all animals was monitored every 2 weeks. After the treatment, the animals were euthanized by exposure to CO_2_ for 10 min (5 min of induction time with CO_2_ flowrate at 20% of the chamber volume per minute and 5 min of dwell time), and tissues were collected for further studies.

### 2.4 Quantification of gene expression

Total RNAs were extracted from mouse RPE and liver using TRIzol™ reagent (cat. no. 15596026, Thermo Fisher Scientific, Paisley, Scotland), following the manufacturer’s guidance. cDNA was synthesized, and the mRNA level of individual targeted genes was measured using a SuperScript III reverse transcription kit (cat. no. 18080093, Thermo Fisher Scientific, Paisley, Scotland) and the Syber Green kit (cat. no. 4309155, Thermo Fisher Scientific, Paisley, Scotland), respectively. The cycle threshold (CT) values of targeted genes were normalized to the housekeeping gene, *Gapdh*, and then analyzed using the 2**^-^** ^ΔΔCt^ formula. Primer sequences are shown in **Supplementary Table S1**.

### 2.5 Biochemical assays

Total cholesterol in mouse RPE, liver, and serum was measured using a commercial cholesterol assay kit (cat. no. A12216, Thermo Fisher Scientific, Paisley, Scotland) based on the manufacturer’s protocol. Proinflammatory cytokines interleukin 1β (IL-1β) and IL-6 and tumor necrosis factor α (TNFα) were detected using the Mini ABTS ELISA Development Kits (cat. no. 900-K47 for IL-1β; cat. no. 900-K50 for IL-6; cat. no. 900-K54 for TNFα; purchased from Thermo Fisher Scientific, Paisley, Scotland), following the manufacturer’s guidance.

### 2.6 Sequencing and bioinformatic analysis of 16S rRNA genes

Animal cecum samples were collected, and total DNA was extracted using a QIAamp DNA Stool Mini Kit (Qiagen, UK) based on the manufacturer’s guidance. The V3+V4 region of the 16S rRNA gene was amplified by PCR using upstream and downstream primers 338F 5′-ACTCCTACGGGAGGCAGCA-3′ and 806R 5′-GGACTACHVGGGTWTCTAAT-3′. The amplified products were separated by agarose gel electrophoresis and purified using a gel extraction kit. The library was constructed by a second PCR with Illumina adapters attached to the originally amplified products. High-throughput sequencing of the library was then conducted using the Illumina NovaSeq 6000 platform.

Raw data were initially filtered using Trimmomatic v0.33, followed by primer sequence removal with Cutadapt v1.9.1. Sequence assembly and chimera removal were performed using USEARCH v10, and reads were clustered into operational taxonomic units (OTUs) at a 97.0% similarity threshold. Alpha diversity was compared using the Simpson and Shannon indices with the R v3.1.1 software. Beta diversity differences between groups were compared using PCoA analysis based on Bray–Curtis and weighted UniFrac distances, performed with QIIME2 software. The relative abundance means of the phyla was visualized by Stacked plots chart, with the cluster of these phyla represented by a heatmap using the SRplot tool (https://www.bioinformatics.com.cn). The differences of the taxa relative abundance between groups were assessed using Wilcoxon tests. Predicated functional profile of the gut microbiome was performed using the Phylogenetic Investigation of Communities by Reconstruction of Unobserved States (PICRUSt2). The differences of the relative abundance of MetaCyc pathways among groups were determined using Wilcoxon tests. In addition, the predicated abundance of functional genes was analyzed based on Kyoto Encyclopedia of Genes and Genomes (KEGG) orthologs (KOs) using 16S rRNA gene sequence database identifiers by KEGG. The relative abundance of KOs across the three experimental groups was assessed using the online iDEP (https://bioinformatics.sdstate.edu/idep/). Differences in KO profiles among the three groups were presented through cluster heatmap, dendrograms, and volcano plot to highlight statistically significant variations compared by log_2_ FC and adjusted *p*-value.

### 2.7 Data analysis

Data from the quantification of gene expression and biochemical assays was presented as mean ± SE and analyzed with the GraphPad Prism software (version 10, www.graphpad.com). *P*-value <0.05 was considered significant.

## 3 Results

### 3.1 Identification of main compounds in water-extracted MZP

**Figure 1:**
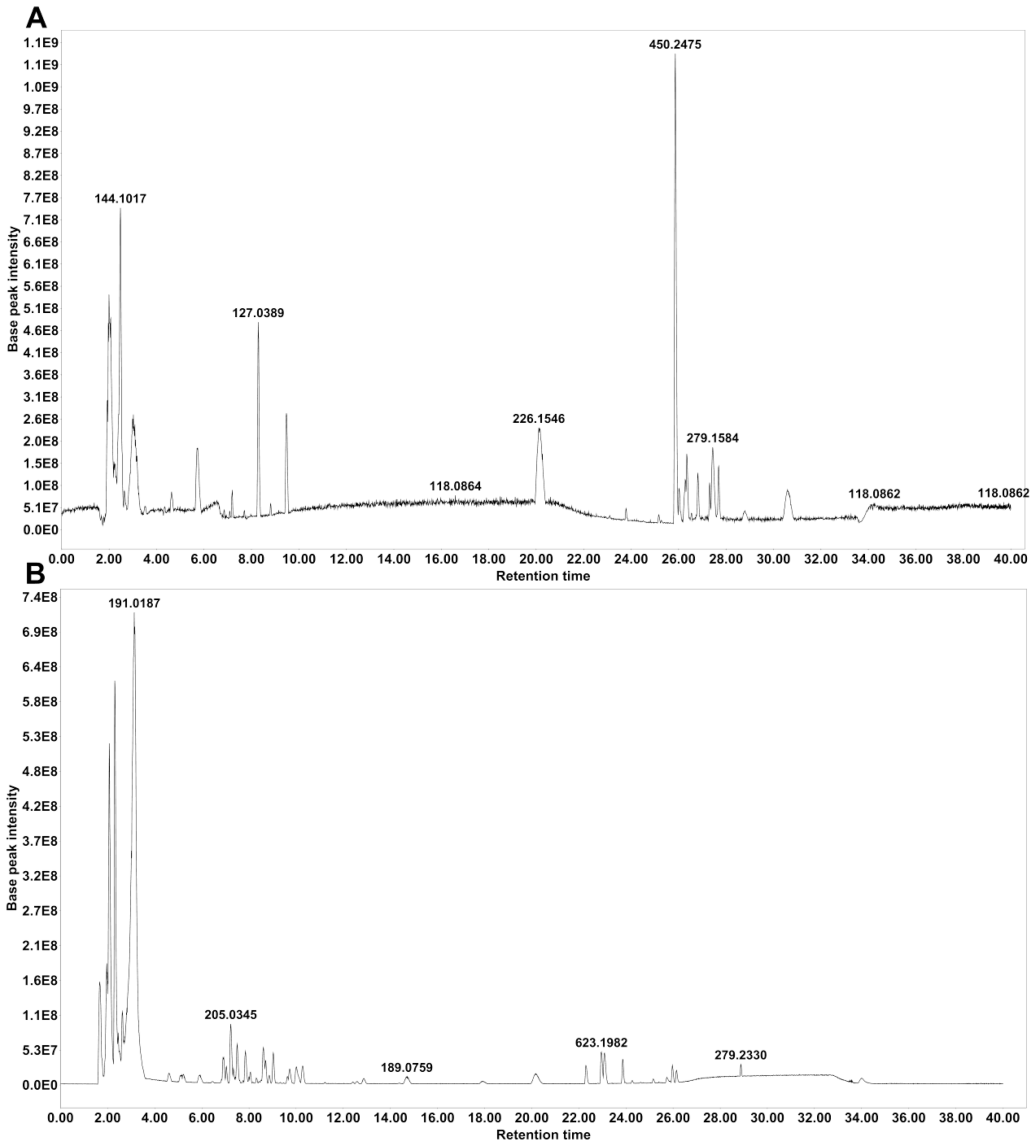
Base peak chromatograms of modified Zhujing pill (MZP) water extract obtained in positive **(A)** and negative **(B)** ionization modes.

HPLC–MS/MS was used to identify the main compounds in water-extracted MZP, and both positive and negative ion modes were analyzed in the total ion current chromatograms (**Figure 1**). Based on the MZmine software, 63 compounds were identified in positive ion mode (**Supplementary Table S2**), and 81 compounds were identified in negative ion mode (**Supplementary Table S3**), when aligning to the two available databases (MASSBANK and GNPS). When the duplicated compounds in both positive and negative ion modes were excluded, 115 compounds remained, of which 23% were flavonoids, 11% were terpenes, 11% were phospholipids, 11% were carboxylic acids, 7% were cinnamic acids, 7% were phenolic, 5% coumarins, 3% pyridines, 3% sesquiterpenes, 3% fatty acids, and 16% were other compounds.

### 3.2 MZP treatment decreased the cholesterol level in the RPE, liver, and serum of HFD-fed mice

The HFD-fed mouse model has been widely used to study the pathogenesis of AMD (Biswas et al., 2021; Keeling et al., 2022). We monitored the body weight of all animals in the three groups during the experimental period and noticed that, from week 2, HFD-fed animals (groups 2 and 3) gained significantly more body weight than that of animals fed with chow diet (group 1). After MZP treatment, the body weight of MZP-treated animals was not significantly different to that of HFD-fed animals, indicating that MZP had no effect on mouse body weight (**Supplementary Figure S2**).

**Figure 2:**
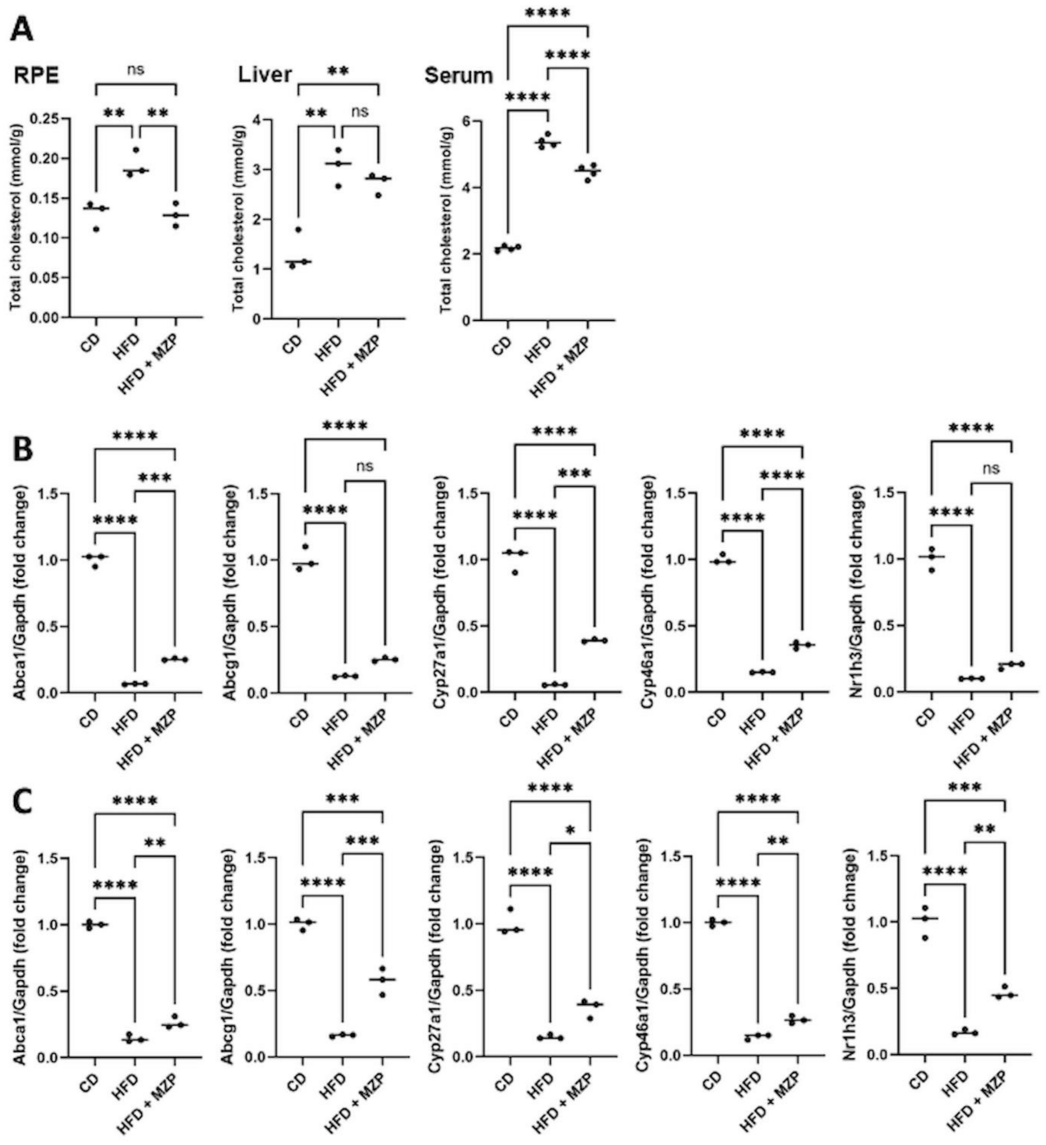
MZP regulated cholesterol metabolism in HFD-fed mice. **(A)** Cholesterol level in the RPE, liver, and serum of three animal groups. Samples from six animals of each group were pooled and subjected to cholesterol measurement. **(B)** mRNA levels of cholesterol trafficking and metabolism genes in the RPE of CD, HFD, and HFD+MZP animals. Samples from six animals of each group were pooled; total RNAs were extracted and subjected to qRT-PCR detection. **(C)** mRNA levels of cholesterol trafficking and metabolism genes in the liver of CD, HFD, and HFD+MZP animals. Samples from six animals of each group were pooled; total RNAs were extracted and subjected to qRT-PCR detection. CD, control diet; HFD, high-fat diet; HFD+MZP, high-fat diet (HFD) + modified Zhujing pill (MZP); RPE, retinal pigment epithelial cells. Ns, no significance; *p < 0.05, **p < 0.01, ***p < 0.001, ****p < 0.0001.

When we examined the cholesterol level in the RPE, liver, and serum in all animals, we found that HFD-fed animals had significantly higher levels of cholesterol in the examined tissues, when compared to that of animals fed with chow diet, and that MZP administration counteracted HFD-induced effects (**Figure 2A**). We also measured the expression of cholesterol homeostasis-associated genes in the RPE and liver. The expression of cholesterol metabolism genes (*Cyp27a1* and *Cyp46a1*), cholesterol metabolism regulator gene (*Nr1h3*, encoding LXRα), and cholesterol trafficking genes (*Abca1* and *Abcg1*) was significantly downregulated in the RPE and liver of HFD-fed mice compared to that of mice fed with chow diet; MZP reversed the HFD-induced effects by upregulating the expression of these genes (**Figures 2B, C**).

### 3.3 MZP treatment modulated the expression of antioxidant and inflammation genes in the tissues of HFD-fed mice

**Figure 3:**
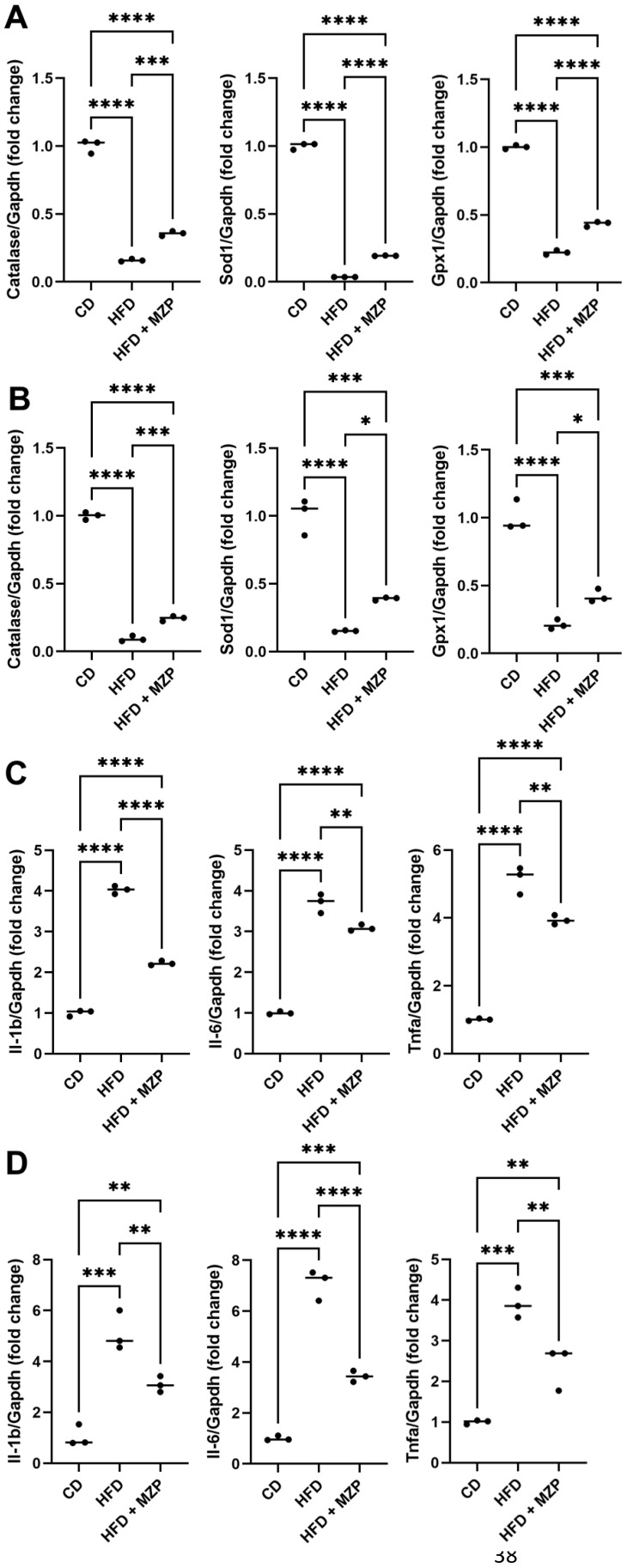
MZP regulated oxidative stress and inflammation in the RPE and the liver. **(A)** mRNA levels of antioxidant genes in the RPE. **(B)** mRNA levels of antioxidant genes in the liver. **(C)** mRNA levels of proinflammatory cytokine genes in the RPE. **(D)** mRNA levels of proinflammatory cytokine genes in the liver. Samples from six animals of each group were pooled; total RNAs were extracted and subjected to qRT-PCR detection. CD, control diet; HFD, high-fat diet; HFD+MZP, high-fat diet (HFD) + modified Zhujing pill (MZP); RPE, retinal pigment epithelial cells. *p < 0.05, **p < 0.01, ***p < 0.001, ****p < 0.0001.

It is well documented that HFD can induce oxidative stress and inflammation, which contribute to the pathogenesis of chronic disorders, including obesity, diabetes, cardiovascular diseases, and neurodegenerative diseases (27). We found that the expression of antioxidant genes *catalase*, *superoxide dismutase* (*Sod1*), and *glutathione peroxidase 1* (*Gpx1*) was significantly decreased in the RPE and liver of HFD-fed mice compared to that of mice fed with chow diet. MZP treatment led to an increase in the expression of these genes, though the expressional levels of the three genes were still less than that of mice fed with chow diet (**Figures 3A, B**). We further examined the expression of proinflammatory cytokine genes IL-1β, IL-6, and TNFα in the RPE and liver of the three groups. HFD-fed mice had a marked increase in the expression of IL-1β, IL-6, and TNFα in the RPE and liver when compared to that of mice fed with chow diet; however, MZP administration significantly decreased the expression of the three genes in both RPE and liver compared to that of HFD-fed animals (**Figures 3C, D**).

**Figure 4:**
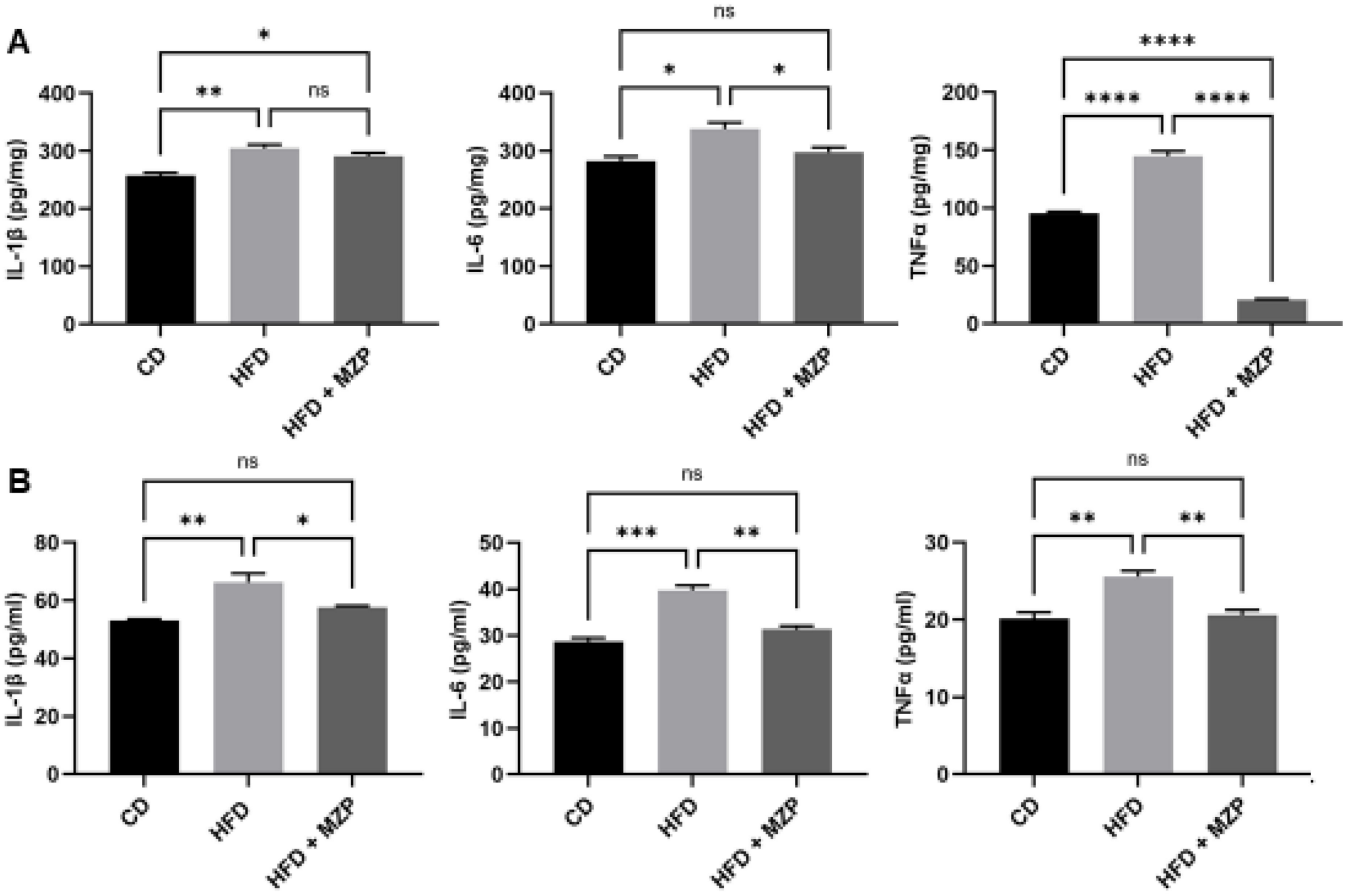
MZP decreased the levels of proinflammatory cytokines in the liver **(A)** and the serum **(B)**. Samples from six animals of each group were pooled and subjected to enzyme-linked immunosorbent assay (ELISA). CD, control diet; HFD, high-fat diet; HFD+MZP, high-fat diet (HFD) + modified Zhujing pill (MZP). Ns, no significance; *p < 0.05, **p < 0.01, ***p < 0.001, ****p < 0.0001.

We also measured the IL-1β, IL-6, and TNFα protein levels in the liver and serum and found that HFD-fed mice had significantly higher levels of the three cytokines than that of mice fed with chow diet. The MZP treatment resulted in a marked decrease in the levels of these cytokines compared to that of HFD-fed mice (**Figure 4**).

### 3.4 MZP administration modulated the diversity and composition of gut microbiota

Numerous studies have reported that HFD induces dysbiosis, which plays an important role in the formation and progression of chronic diseases (Chen et al., 2023). We analyzed the microbiota in the cecum of six animals of each group using QIIME 2 Software. In total, 1,474,934 sequence reads was obtained from all samples. Following quality control filtering, overlapping and merging paired-end reads for each individual sample, removing reads that failed to merge, and chimera filtering, 1,161,226 high-quality non-chimeric merged reads were used for bacterial taxonomic and diversity analysis (**Supplementary Table S4**).

**Figure 5:**
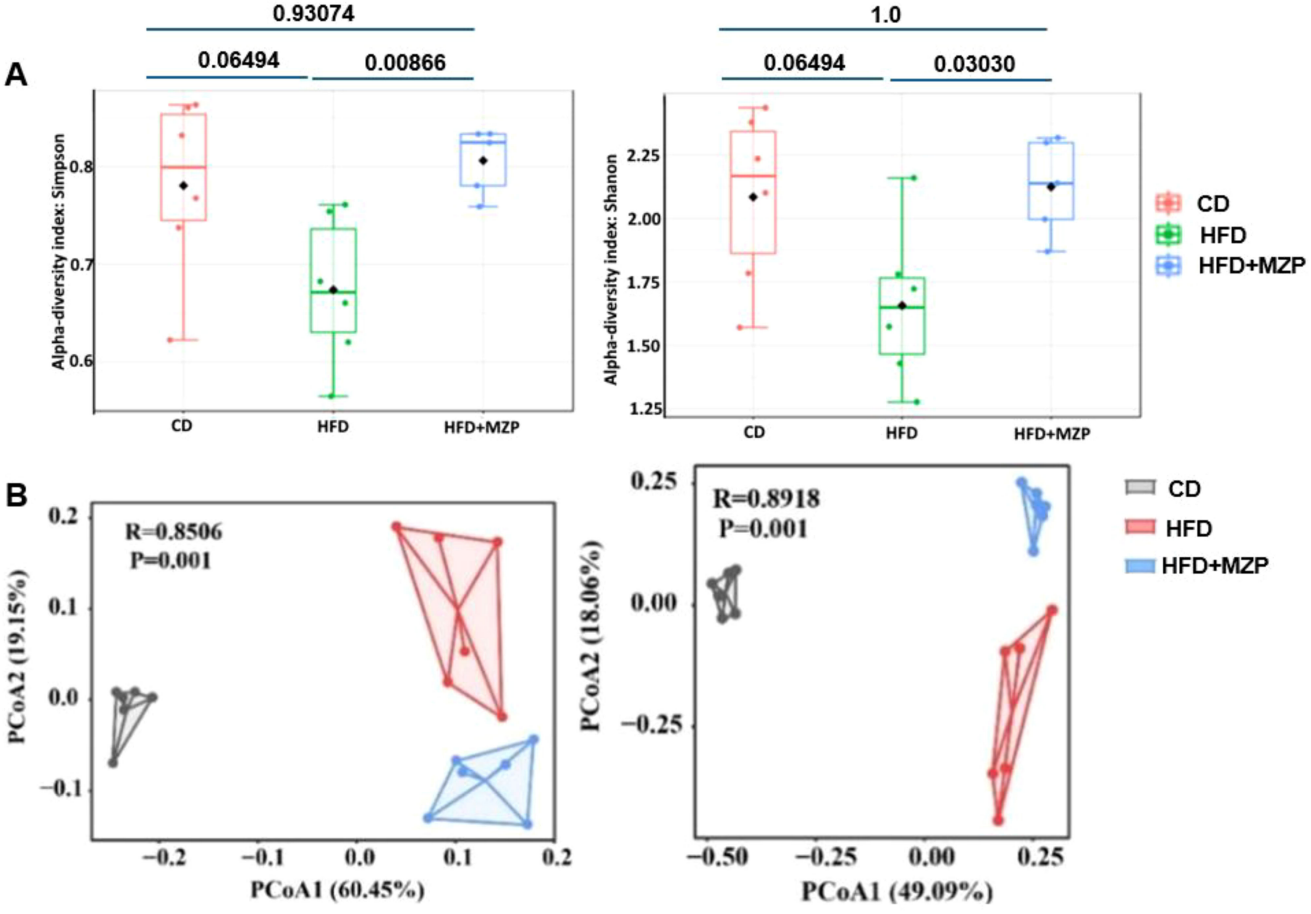
**(A)** α-diversity pf bacterial communities among the three animal groups using the Simpson and Shannon index. **(B)** β-diversity of bacterial communities among the three animal groups using Bray–Curtis (left panel) and weighted unifrac (right panel) index. CD, control diet; HFD, high-fat diet; HFD+MZP, high-fat diet (HFD) + modified Zhujing pill (MZP).

Alpha (α) diversity measures the diversity of microbial communities in individual samples, while beta (β) diversity refers to the difference in composition of microbial communities between samples. We applied α- and β-diversities to analyze the diversity and composition of the cecum bacterial communities in the three groups. A general reduction in the α-diversity metrics was observed in the HFD group compared to the CD group, though there was no significant difference (**Figure 5A**); on the other hand, the metrics in the MZP-treated group were significantly increased when compared to the HFD group, nearly returning to the level of the CD group. PCoA of β diversity analysis based on the unweighted UniFrac distance matrix (PCo1 contribution 60.45%, PCo2 contribution 19.15%) and the Bray–Curtis distance matrix (PCo1 contribution 49.09%, PCo2 contribution 18.06%) revealed that samples from the CD and HFD + MZP groups were closely clustered, while samples from the HFD group were more dispersed (**Figure 5B**). This suggests a higher similarity in microbiota composition within the CD and HFD + MZP groups and greater variability within the HFD group. These findings indicated that MZP has a potential regulatory effect on the gut microbiota structure altered by a high-fat diet.

**Figure 6:**
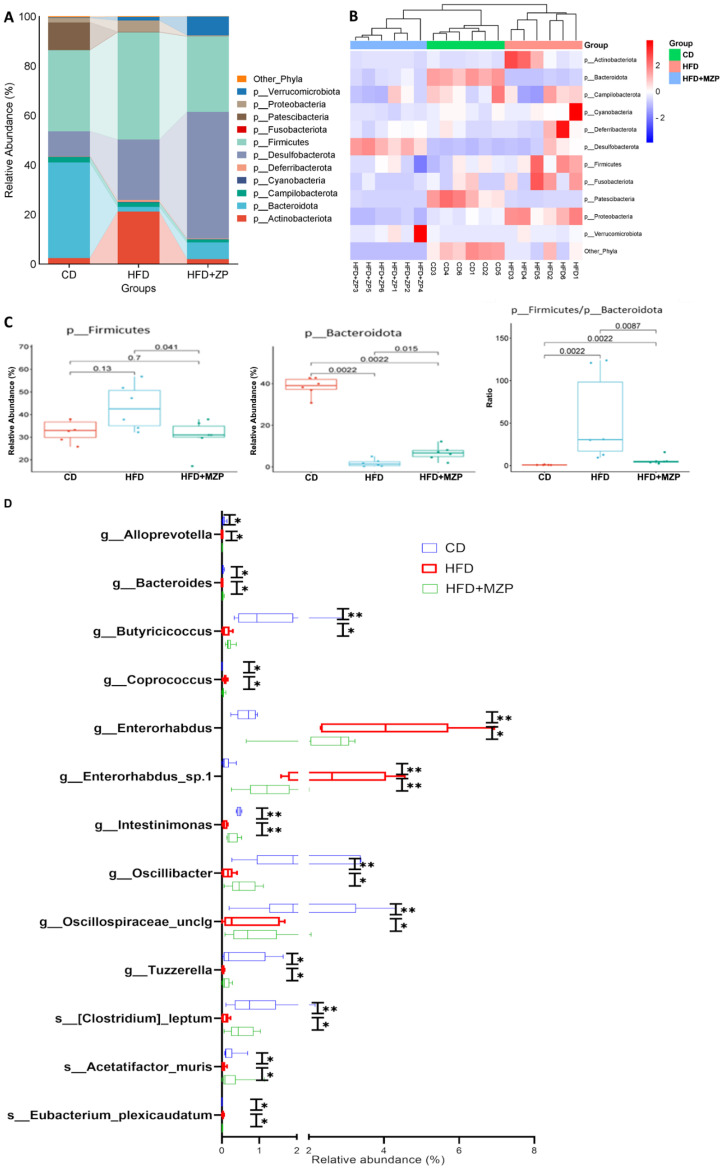
MZP modulated cecum microbiota. **(A)** Different abundance of the phyla among the three experimental groups. **(B)** Heatmap representation of phyla abundance from individual samples of three experiment groups. **(C)** Comparison of the abundance of *Firmicutes* and *Bacteroidota* among the three experimental groups and the ratio of *Firmicutes*/*Bacteroidota*. **(D)** Genera and species with significantly increased or decreased abundance in the HFD group, when compared to both the CD and HFD+MZP groups. CD, control diet; HFD, high-fat diet; HFD+MZP, high-fat diet (HFD) + modified Zhujing pill (MZP). *p < 0.05, **p < 0.01.

To analyze the phylogenetic structure and relative abundance of gut microbiota, a total of 205 taxa were identified from 14 phyla, including *Acidobacteriota*, *Actinobacteriota*, *Armatimonadota*, *Bacteroidota*, *Campilobacterota*, *Cyanobacteria*, *Deferribacterota*, *Desulfobacterota*, *Firmicutes*, *Fusobacteriota*, *Patescibacteria*, *Proteobacteria*, *Chloroflexi*, and *Verrucomicrobiota*, containing 63 classified families and 103 classified genera (**Supplementary Table S5**). Notably, the structure and relative abundance of the gut bacteria were significantly varied among the three experimental groups. **Figure 6A** illustrates the relative abundance of gut bacteria at the phylum level. In the CD group, *Bacteroidota* and *Firmicutes* were the predominant phyla, followed by *Patescibacteria* and *Desulfobacterota*; in the HFD group, *Firmicutes* was the predominant phylum, followed by *Desulfobacterota* and *Actinobacteriota*. By contrast, in the MZP-treated group, *Desulfobacterota* was the predominant phylum, followed by *Firmicutes*. Heatmap analysis also demonstrated distinct clustering patterns among the three groups, indicating a clear separation of the gut bacterial profiles of the HFD group from the same cluster within the branch for both CD and MZP-treated groups (**Figure 6B**). *Firmicutes* and *Bacteroidota* are the two major bacterial phyla in the gut and play a critical role in maintaining gut health. HFD can dysregulate the gut microbiota, mainly the abundance of *Firmicutes* and *Bacterodota* (Chen et al., 2023). We also found that *Firmicutes* in the HFD group was significantly more abundant in the HFD group compared to both the CD and HFD+MZP groups, while the abundance of *Firmicutes* in the CD group was not significantly different to that of the HFD+MZP group. However, the abundance of *Bacteroidetes* was marked lower in the HFD group compared to that of the CD group, while the abundance of *Bacteroidetes* was significantly increased in the HFD+MZP group compared to that of the HFD group. The ratio of *Firmicutes*/*Bacteroidetes*, a biomarker for dysbiosis, was significantly higher than that of both the CD and HFD+MZP groups (**Figure 6C**). In addition, HFD induced a significant elevation of the abundance in both *Actinobacteriota* and *Proteobacteria* phyla, and MZP reversed the HFD-induced effect (data not shown).

Several taxa were markedly increased in the HFD group, but these taxa were significantly reduced in the HFD+MZP group, with levels partially restoring toward those observed in the CD group. These taxa were g_*Alloprevotella*, g_*Coprococcus*, g_*Enterorhabdus*, g_*Enterorhabdus*_sp.1, and s_*Eubacterium*_*plexicaudatum*. By contrast, some taxa that were reduced in the HFD group showed an increased abundance in the HFD+MZP group, resembling more closely the CD group. These included g_*Bacteroides*, g_*Butyricicoccus*, g_*Intestinimonas*, g_*Oscillospiraceae_unclg*, g_*Tuzzerella*, s_*Acetatifactor_muris*, and s:[*Clostridium*]_*leptum* (**Figure 6D**). In addition, various other taxa exhibited differential abundance patterns among the three groups, indicating broader microbial shifts influenced by dietary intervention and MZP treatment (data not shown).

### 3.5 MZP moderated the metabolic pathways of bacterial communities

**Figure 7:**
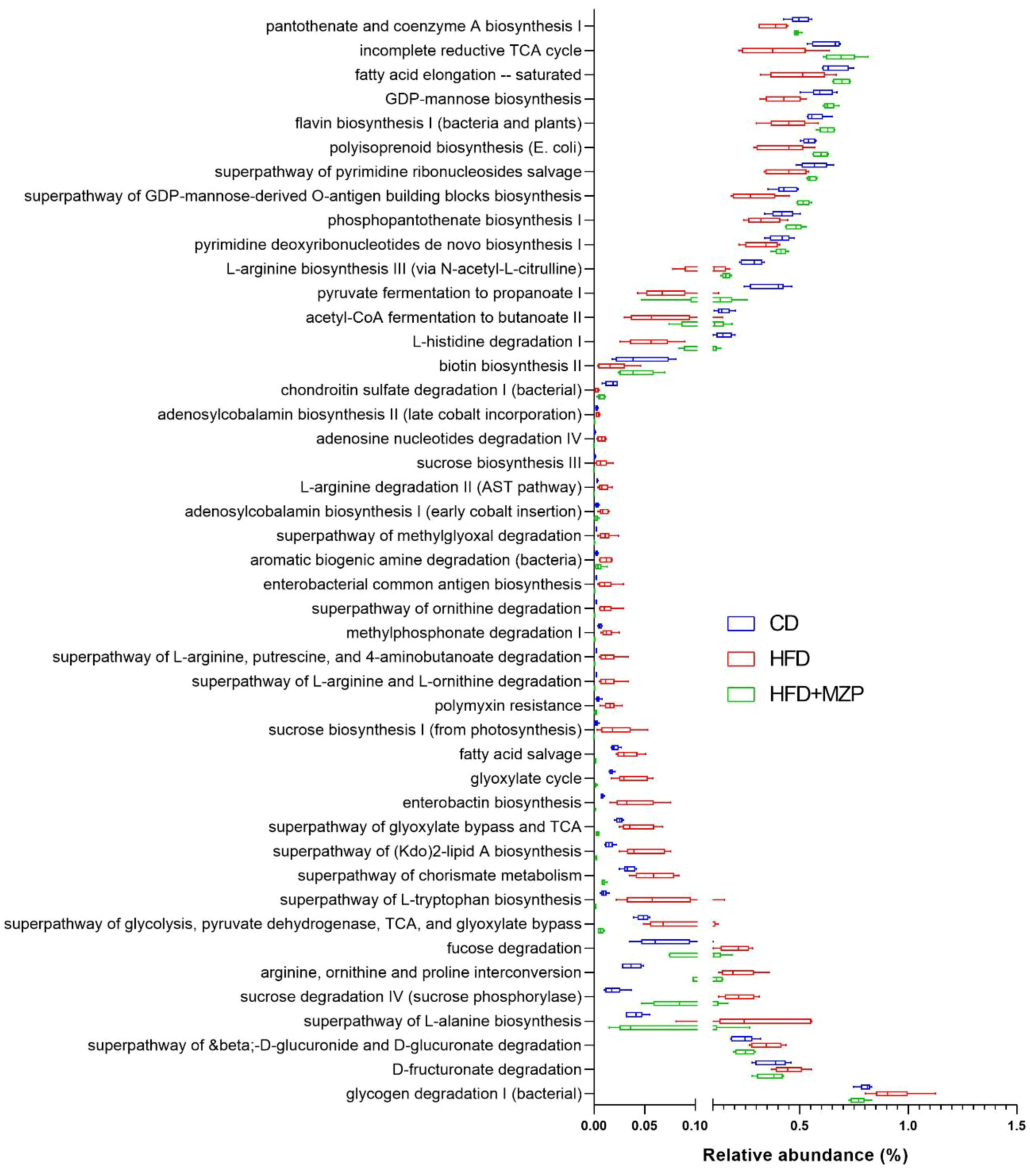
MetaCyc pathways with significantly decreased or increased relative abundance in the HFD group when compared to both the CD and HFD+MZP groups. CD, control diet; HFD, high-fat diet; HFD+MZP, high-fat diet (HFD) + modified Zhujing pill (MZP).

PICRUSt2 was carried out to determine the MetaCyc metabolic pathways to explore the roles of gut microbiome across the three experimental groups. Out of the 393 predicted MetaCyc pathways, 235 were significantly different among the three experimental groups (**Supplementary Table S6**) after excluding those pathways with a mean relative abundance less than 0.001 in all groups. When comparing the HFD group to the CD group, the abundance of 55 metabolic pathways was significantly increased and 18 pathways decreased; when comparing the HFD group to the HFD+MZP group, the abundance of 93 pathways was significantly increased and 51 pathways decreased; when comparing the HFD+MZP group to the CD group, the abundance of 75 pathways was significantly increased and 100 pathways decreased (**Supplementary Table S6**). Notably, the abundance of 16 pathways—for example, flavin biosynthesis I, acetyl-CoA fermentation to butanoate II, and biotin biosynthesis II—in the HFD group was significantly lower than that of both the CD and HFD+MZP groups; 29 pathways—e.g., enterobacterial common antigen biosynthesis and superpathway of (Kdo)2-lipid A biosynthesis—were significantly enriched in HFD when compared to both the CD and HFD+ZP groups (**Figure 7**). The data indicated a strong functional shift induced by a high-fat diet alone and restoration in the MZP-treated group to the CD group.

**Figure 8:**
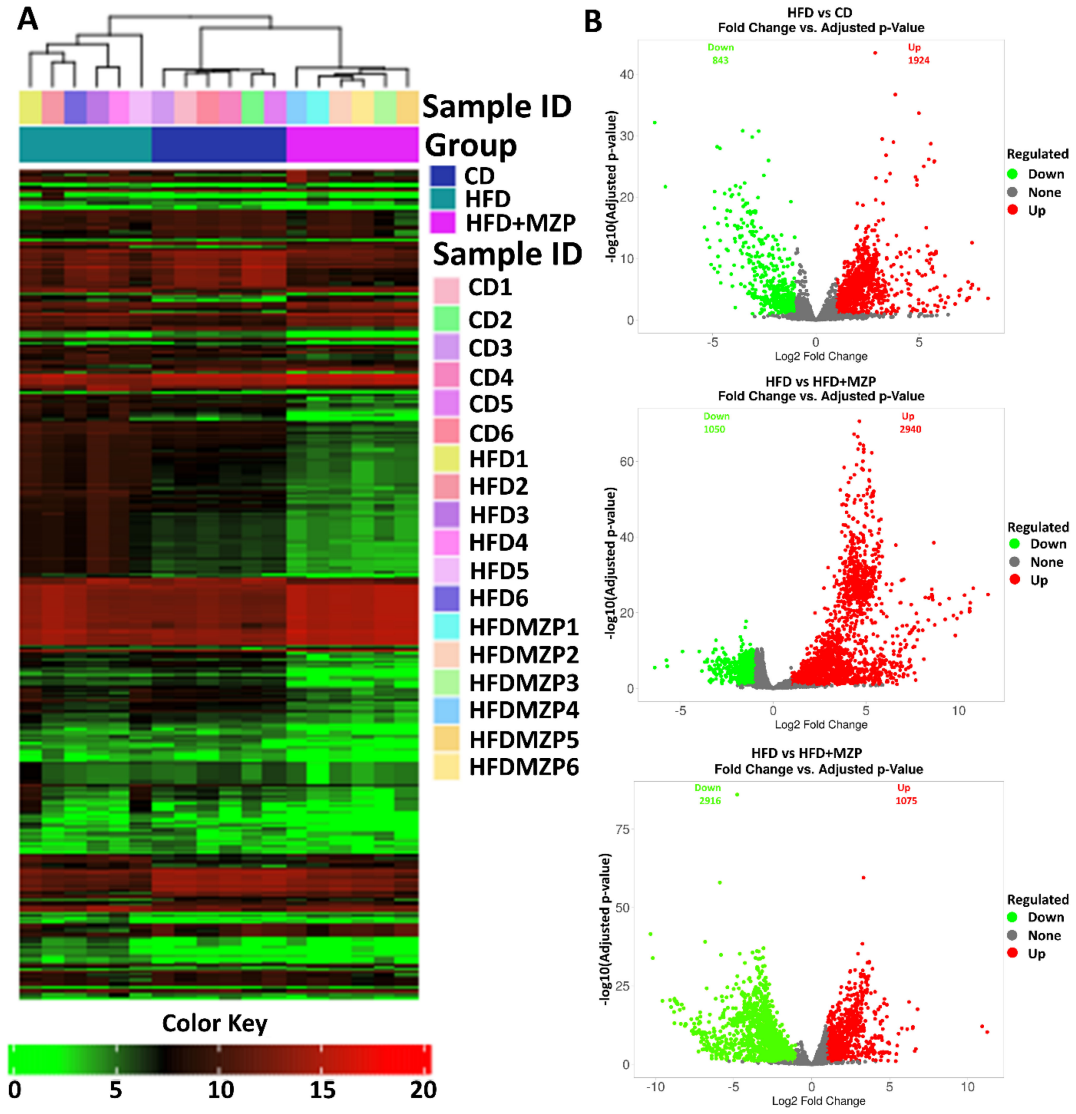
MZP regulated the abundance of Kyoto Encyclopedia of Genes and Genomes orthologs (KOs). **(A)** Heatmap representation of KOs in individual samples of the three experimental groups. **(B)** Volcano plots showing upregulated (labeled in red) and downregulated (labeled in green) KOs, compared between two groups. CD, control diet; HFD, high-fat diet; HFD+MZP, high-fat diet (HFD) + modified Zhujing pill (MZP).

**Figure 9:**
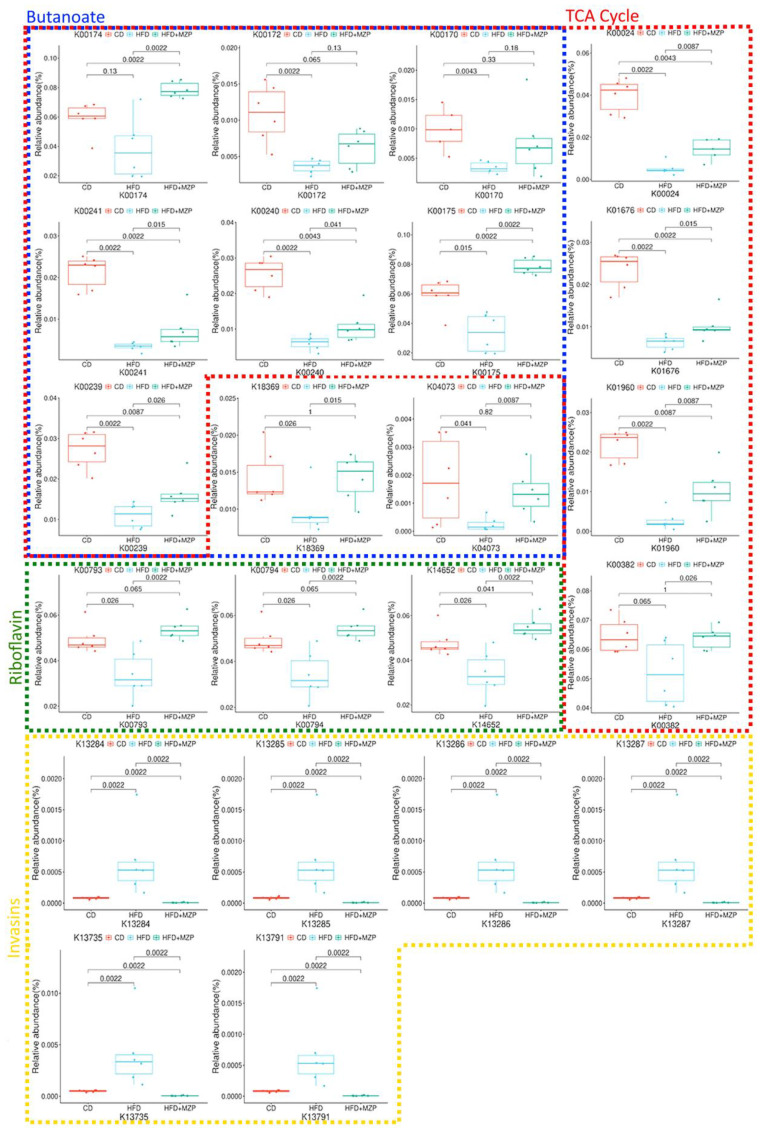
Selected KOs involved in citrate cycle, butanoate metabolism, riboflavin metabolism, and invasion signaling. CD, control diet; HFD, high-fat diet; HFD+MZP, high-fat diet (HFD) + modified Zhujing pill (MZP).

In addition, bacterial functional gene content based on the KEGG orthologs (KOs) was predicted using PICRUSt2 to highlight the key roles of the gut microbiome across three experimental groups. From a total of 6,433 predicted KOs, the top 4,300 most abundant KOs were selected for further analysis. Heatmaps and hierarchical clustering visualization of those KOs across the 18 samples revealed a distinct separation between the HFD group and both the CD and HFD+MZP groups, using absolute Pearson distance with complete linkage clustering (**Figure 8A**). Additional analysis using volcano plots was conducted to assess differential KO abundance based on log_2_ fold changes and adjusted *p*-value (-log_10_). **Figure 8B** illustrates that the abundance of 843 KOs was significantly reduced and that of 1,924 was increased in the HFD group compared to the CD group; similarly, when comparing the HFD group to the HFD+MZP group, the activity of 1,050 KOs was significantly reduced and that of 2,940 was elevated. Conversely, the abundance of 2,916 KOs was downregulated and that of 1,075 upregulated when the HFD+MZP group was compared to the CD group. **Supplementary Figure S3** highlights the top 50 significantly altered KOs (25 upregulated and 25 downregulated) in the HFD group compared to the HFD+MZP group. These KOs were also used for comparison among the other experimental groups, indicating consistent functional shifts by MZP treatment. Among the significantly changed KOs, functions associated with citrate cycle (tricarboxylic acid cycle), butanoate metabolism, and riboflavin metabolism were significantly decreased in the HFD group compared to both the CD and HFD+MZP groups. By contrast, entries associated with the invasion signaling pathway were markedly abundant in the HFD group compared to both the CD and HFD+MZP groups (**Figure 9**).

### 3.6 Cecum bacteria were correlated with proinflammatory cytokines

**Figure 10:**
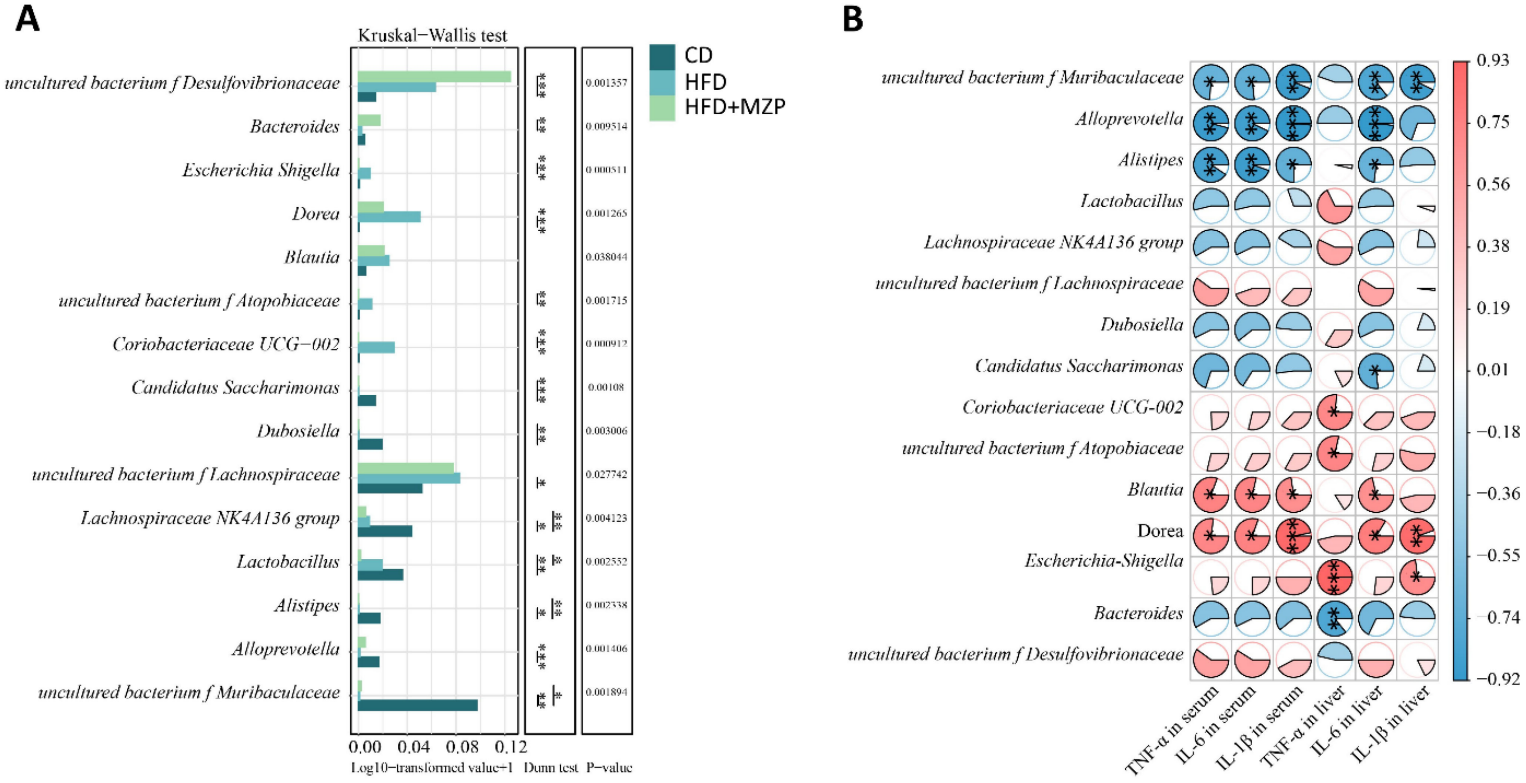
Correlation analysis between cecum characteristic genera and proinflammatory cytokines. **(A)** Multifunctional differential analysis of characteristic genera. **(B)** Heatmap showing the correlation between liver and serum proinflammatory cytokines and bacteria. Mann–Kruskal–Wallis followed by Dunn’s multiple-comparisons test was used to estimate the significant differences. The *t*-test based on the Pearson correlation coefficient was used to test the significance of the correlation coefficient. *p < 0.05; **p < 0.01; ***p < 0.001 (*n* = 6). CD, control diet; HFD, high-fat diet; HFD+MZP, high-fat diet (HFD) + modified Zhujing pill (MZP).

The results of the correlation analysis between cecum bacterial and proinflammatory cytokines are presented in **Figure 10**. The data indicated that the relative abundance of the characteristic bacterial genera *uncultured bacterium f Muribaculaceae* and *Candidatus Saccharimonas* in the CD group showed a significant negative correlation with liver IL-6 level (*p* < 0.05). Additionally, the relative abundance of *uncultured bacterium f Muribaculaceae*, *Alloprevotella*, and *Alistipes* was significantly negatively correlated with the serum levels of IL-1β, IL-6, and TNF-α (*p* < 0.05).

The relative abundance of the characteristic bacterial genera *Coriobacteriaceae UCG-002*, *uncultured bacterium f Atopobiaceae*, and *Escherichia Shigella* in the HFD group exhibited a significant positive correlation with the liver TNF-α level (*p* < 0.05). Additionally, the relative abundance of *Blautia* and *Dorea* was significantly positively correlated with the liver IL-6 levels as well as serum levels of IL-1β, IL-6, and TNF-α (*p* < 0.05).

The relative abundance of the characteristic bacterial genus *Bacteroides* in the HFD+MZP group was negatively correlated with the levels of IL-1β, IL-6, and TNF-α in both the liver and serum, while the relative abundance of u*ncultured bacterium f Desulfovibrionaceae* was positively correlated with the levels of those cytokines. Notably, the liver TNF-α level showed a significant negative correlation with the relative abundance of *Bacteroides (p* < 0.01).

## 4 Discussion

Although MZP is widely used to treat AMD patients and has shown therapeutic benefits, the protective mechanisms are poorly understood. Here we treated HFD-fed mice, a commonly used AMD model, with MZP for 4 weeks and found that MZP counteracted the HFD-induced effects via decreasing the cholesterol accumulation, upregulating the expression of cholesterol metabolism and trafficking genes, and suppressing the oxidative stress and inflammation in mouse RPE and liver as well as modulating the composition, diversity, and metabolic pathways of gut microbiota.

Cholesterol is a versatile lipid with multiple functional roles, such as maintaining cell membrane structure, producing hormones, assisting digestion, and regulating cell signaling pathways. Cholesterol biosynthesis, metabolism, and excretion are tightly controlled, and dysregulation of cholesterol homeostasis is associated with various disorders, e.g., cardiovascular and neurodegenerative diseases (Duan et al., 2022). Abnormal deposits of cholesterol and its metabolites (e.g., 7-ketocholesterol) are enriched in the drusen of AMD patients, suggesting that cholesterol plays an important role in the pathogenesis of AMD (Zhang et al., 2021). The RPE has a high-level expression of cholesterol homeostasis-associated genes, including *HMGCR, SCREBP2, SCAP, NR1H3, CYP27A1, CYP46A1, ABCA1,* and *ABCG1* (Zheng et al., 2015; Zhang et al., 2021). Global loss of mouse cholesterol metabolism enzyme CYP27A1 or CYP46A1 results in dysregulated retinal cholesterol homeostasis, sub-RPE deposits of cholesterol, and abnormal vascularization in the retina (Omarova et al., 2012; Saadane et al., 2019). Conditional loss of both cholesterol transporters ABCA1 and ABCG1 in the RPE causes intracellular abnormal lipid accumulation composed predominantly of cholesteryl esters in the RPE, RPE atrophy, and progressive photoreceptor degeneration (Storti et al., 2019). Here we found that HFD, which is associated with an increased risk of developing AMD in humans, induced the elevated level of cholesterol in the RPE and downregulated the expression of genes related to cholesterol metabolism and transport. The downregulated expression of these genes can lower cholesterol metabolism and trafficking, resulting in the accumulation of cholesterol in the RPE. MZP treatment lowered the cholesterol level in HFD-fed mouse RPE, possibly via promoting cholesterol metabolism and trafficking. Excess cholesterol in the RPE returns via reverse transport to the liver, where cholesterol can be stored as cholesteryl esters or excreted by forming bile acids. HFD also increased the cholesterol level in the liver; one possible explanation is a deficit in bile acid biosynthesis, as the expression of *Cyp27a1*, one of the bile acid synthesizing enzymes, was downregulated, and MZP counteracted the effect. Therefore, it would be worth exploring the bile-acid-synthesis-associated enzymes in the liver of experimental animals.

HFD, known as “Western diet”, can induce oxidative stress and inflammation and is associated with an increased risk of developing AMD (Biswas et al., 2021; Sterling et al., 2022). Rodents fed with HFD demonstrate the pathological features of AMD; for example, HFD intake potentiates laser-induced choroidal neovascularization, a major pathological feature of neovascular AMD (Andriessen et al., 2016; Xiao et al., 2023). A previous work has demonstrated that HFD induces the production of reactive oxygen species (ROS), lowers the expression of antioxidant genes, and upregulates the expression of proinflammatory cytokines in the RPE (Biswas et al., 2021). Here we also found that HFD downregulated the expression of antioxidant genes and upregulated the expression of proinflammatory cytokine genes in the RPE and liver, and MZP treatment reversed the HFD-induced effects. This data is consistent with an earlier report on the antioxidant capacity of MZP in human RPE cells treated with oxidized low-density lipoprotein (Yang et al., 2025). The molecular mechanisms of HFD-induced oxidative stress and inflammation are complicated, involving mitochondrial dysfunction, ROS production, activation of proinflammatory signaling, and damages to cells and tissues. One of the possibilities is associated with cholesterol, as accumulated cholesterol in the RPE can be autoxidized to form toxic oxysterols, such as 7-ketocholesterol, which are enriched in the drusen and can induce oxidative stress, inflammation, and damage to the RPE and photoreceptor cells (Zhang et al., 2021). Additionally, a recent work has shown that HFD causes damage to RPE cells via IL-1β-regulated expression of iron importers and exporters, iron accumulation, and oxidative damage (Sterling et al., 2022). Therefore, it is reasonable to investigate further whether MZP treatment decreases the production of toxic oxysterols and regulates the expression of iron importers/exporters in the RPE of HFD-fed mice.

Accumulated data have demonstrated that abnormal alteration of the gut microbiota is associated with AMD. A previous report by Zinkernagel et al. showed that AMD patients had abundant genera *Anaerotruncus* and *Oscillibacter* together with species *Ruminococcus torques* and *Eubacterium ventriosum* and low abundance of species *Bacteroides eggerthii* when compared to that of healthy controls. Metabolic functional analysis predicted that the abundance of glutamate degradation, L-alanine fermentation, and arginine biosynthesis was higher in AMD patients (Zinkernagel et al., 2017). Another study reported that AMD patients had a greater abundance of genera *Lactobacillus* and *Veillonella* and a decreased abundance of genera *Anaerobutyricum*, *Anaerosipes*, *Blautia*, *Desulfovibrio*, *Eggerthella*, *Faecalibacterium*, *Massilistercora*, and *Megamonas*. The authors also identified increased activities in 15 metabolic pathways and decreased activities in 45 metabolic pathways in AMD patients compared to that of the controls (Xue et al., 2023). The composition of the gut microbiota is predominantly influenced by diet. HFD can induce dysbiosis in the hosts and contribute to the pathogenesis of neurodegenerative disorders, including AMD (Cao et al., 2022b; Chen et al., 2023). A previous study showed that HFD resulted in a decreased abundance of *Bacteroidetes* and an increased abundance of *Firmicutes* as well as an elevated ratio of *Firmicutes*/*Bacteroidetes* compared to that of chow diet; HFD also induced high levels of gut permeability, lipopolysaccharide (LPS) production, and systemic inflammation, which possibly exacerbated laser-induced choroidal neovascularization, a key pathological feature of neovascular AMD (Andriessen et al., 2016). In the current study, we also found significant changes in the composition of microbiota in the three experimental groups. HFD-fed animals had an increased abundance of *Firmicutes* and a decreased abundance of *Bacteroidota* with a higher ratio of *Firmicutes*/*Bacteroidota* as well as abundance changes for some genera and species; MZP treatment partially reversed those changes back to similar levels of the CD group.

Gut microbiota has been reported to play an important role in cholesterol metabolism via cholesterol conversion. Some gut bacteria can directly convert cholesterol to coprostanol, which is poorly absorbed by the intestine and leads to an increase in cholesterol excretion into the feces and a reduction of cholesterol absorption into the bloodstream (Kenny et al., 2020). Kenny et al. identified the bacterial cholesterol-metabolizing enzymes, encoded by the intestinal sterol metabolism A (ismA) genes in *Clostridium* cluster IV-related bacteria, and found that individuals with high levels of IsmA-encoding bacteria are associated with low levels of fecal and serum cholesterol (Kenny et al., 2020). Li et al. further demonstrated that the abundance of *Oscillibacter* clusters was associated with low levels of fecal and serum cholesterol and identified genes encoding cholesterol-α-glucosyltransferase (CgT), IsmA, and TSPO. CgT is known to metabolize cholesterol to cholesteryl glucosides, IsmA converts cholesterol to coprostanol, and TSPO transports cholesterol from the mitochondrial outer membrane to the inner membrane (Li et al., 2024). In the current study, we found that the abundance of *Oscillibacter* genus and *Clostridium leptum* was significantly lower in the HFD group than that of both CD and HFD+MZP groups. This possibly leads to decreased cholesterol metabolism in the gut and increased uptake of cholesterol into the bloodstream. Our results showed that the HFD group had higher levels of cholesterol in the serum, liver, and RPE compared to that of both the CD and HFD+MZP groups. *Oscillibacter-*encoded TSPO may also play a role in the increased levels of cholesterol in HFD-fed mouse tissues, as our previous studies have shown that the loss of TSPO in mice results in higher levels of cholesterol in the serum, liver, and RPE (Farhan et al., 2021, 2025).

MetaCyc pathway analysis showed significant changes in many metabolic pathways among the experimental groups, of which activities in 16 pathways were significantly decreased and in 29 pathways were significantly increased compared to the CD group. MZP treatment reversed these changes. Some of those pathways—such as the biosynthesis of coenzyme A (CoA) and flavin, acetyl-CoA fermentation to butanoate II (significantly decreased in the HFD group), and superpathway of (kdo)2-lipid A biosynthesis (significantly increased in the HFD group)—are possibly associated with AMD pathogenesis as their functions are related to oxidative stress and inflammation. CoA functions in multiple metabolic pathways, such as citrate cycle, fatty acid oxidation, and synthesis of amino acids. CoA can also play a protective role against oxidative stress through protein CoAlation, which protects cysteine residues being irreversibly oxidized by ROS (Filonenko and Gout, 2023). Furthermore, CoA plays a crucial role in anti-inflammation by modulating immune cell activation and function (Miallot et al., 2023). Flavin is a co-factor for various enzymes, such as glutathione reductase that restores intracellular glutathione; flavin functions in various metabolic pathways, and the deficiency of flavin homeostasis is associated with retina aging and degeneration (Sinha et al., 2018). Butanoate is synthesized by gut bacteria and plays a crucial role in regulating host metabolic pathways and inhibiting inflammation. Previous studies have demonstrated that butanoate can enhance retinal function, suppress retinal inflammation, and mitigate retinal pathology in models of retinal degeneration (Nguyen et al., 2024). Conversely, enterobacterial common antigen, enterobactin, (kdo)2-lipid A, and polymixin are proinflammation-generating factors that can induce inflammation and damage to the host (Holst, 2007; Wang et al., 2015; Saha et al., 2020; Kagi et al., 2022). (kdo)2-lipid A is the core component of LPS, which can activate inflammation via toll-like receptor 4 (TLR4) signaling pathway in the RPE and exacerbate laser-induced neovascularization in mice (Tsioti et al., 2024). Therefore, it would be worthwhile examining these beneficial and toxic bacterial metabolites in host tissues, e.g., the gut, the liver, and the RPE. Further analysis of KOs demonstrated that many pathways were upregulated or downregulated when a comparison was made between the two experimental groups. Some upregulated or downregulated pathways induced by HFD were fully or partially reversed by MZP treatment. Of particular interest is the fact that functions associated with TCA cycle, metabolism of butanoate, and riboflavin were less abundant in the HFD group compared to both the CD and HFD+MZP groups. These pathways are associated with cellular energy production in the mitochondria (Donohoe et al., 2011; Jackson and Theiss, 2020; Vezza et al., 2020), suggesting that there is a crosstalk between gut microbiota and host mitochondrial function and that MZP can possibly enhance the mitochondrial function via modulating the gut microbiota. On the other hand, functions related to the invasin signaling pathway were more abundant in the HFD group compared to the CD and HFD+MZP groups. The invasin signaling pathway can facilitate pathogenic bacterial infection and induce inflammation (Dhar and Virdi, 2014). Therefore, MZP may inactivate the invasion signaling pathway and suppress bacterial infection and associated inflammation.

Analyses of gut microbiota and proinflammatory cytokines showed that some bacterial genera/families were positive or negatively associated with serum and/or liver proinflammatory cytokines. It should be noted that *Dora* was positively associated with serum IL-1β, IL-6, and TNFα and liver IL-1β and IL-6. The abundance of Dora was also significantly higher in the HFD group compared to that of the CD and HFD+MZP groups. Genus *Dorea* has been reported to be associated with an increased risk of developing early AMD (Wei et al., 2025). *Dorea* plays an important role in the formation of gut barrier and demonstrates proinflammatory capacities including the induction of IFNγ, metabolism of sialic acids, and degradation of mucin; patients with inflammatory disorders also have a high abundance of *Dorea* (Shahi et al., 2017). On the other hand, *Alistipes*, *Alloprevotella*, and uncultured bacterium f *Muribaculaceae* were negatively associated with serum IL-1β, IL-6, and TNFα and liver Il-6. The abundance of *Alistipes*, *Alloprevotella*, and uncultured bacterium f *Muribaculaceae* was significantly lower in the HFD group compared to the CD group, and MZP treatment slightly increased the abundance of *Alloprevotella* and *uncultured bacterium f Muribaculaceae* in the HFD-MZP group compared to that of the HFD. The data suggests that *Alistipes*, *Alloprevotella*, and uncultured bacterium f *Muribaculaceae* possibly are associated with anti-proinflammatory effects, consistent with some previous reports (Li et al., 2020; Zhu et al., 2024; Lin et al., 2025).

MZP contains eight medicinal herbs, of which we identified 115 compounds. Most of the identified compounds are polyphenols, including flavonoids (23%), phenolics (7%), and coumarins (5%). Polyphenols have been demonstrated to have beneficial effects against oxidative stress and inflammation in AMD models (Pawlowska et al., 2019). Individual or a combination of polyphenols (luteolin, naringenin, and quercetin, also identified in MZP in the present study) has been shown to suppress H_2_O_2_-induced oxidative stress and inflammation in human RPE cells (Cao et al., 2022a). Previous studies have shown that MZP treatment increased the SOD activity and GSSH-Px level and lowered the levels of proinflammatory effectors in the serum and the retina of diabetic rats (Lei et al., 2018). Here we also found that MZP upregulated the expression of antioxidant genes and downregulated the expression of proinflammatory cytokine genes. In addition, our preliminary data demonstrated that MZP decreased ROS production, increased the expression of antioxidant genes, and alleviated the expression of proinflammatory cytokines in H_2_O_2_-challenged human RPE cells (data not shown). Polyphenol-enriched diet extracts or individual polyphenols have been shown to regulate cholesterol metabolism, modulate the gut microbiota, and provide protective effects in treating cholesterol-associated disorders (Cheng et al., 2023). Future studies will validate the beneficial effects of individual or a combination of identified polyphenols against AMD in *in vitro* and *in vivo* models.

## 5 Conclusion

**Figure 11:**
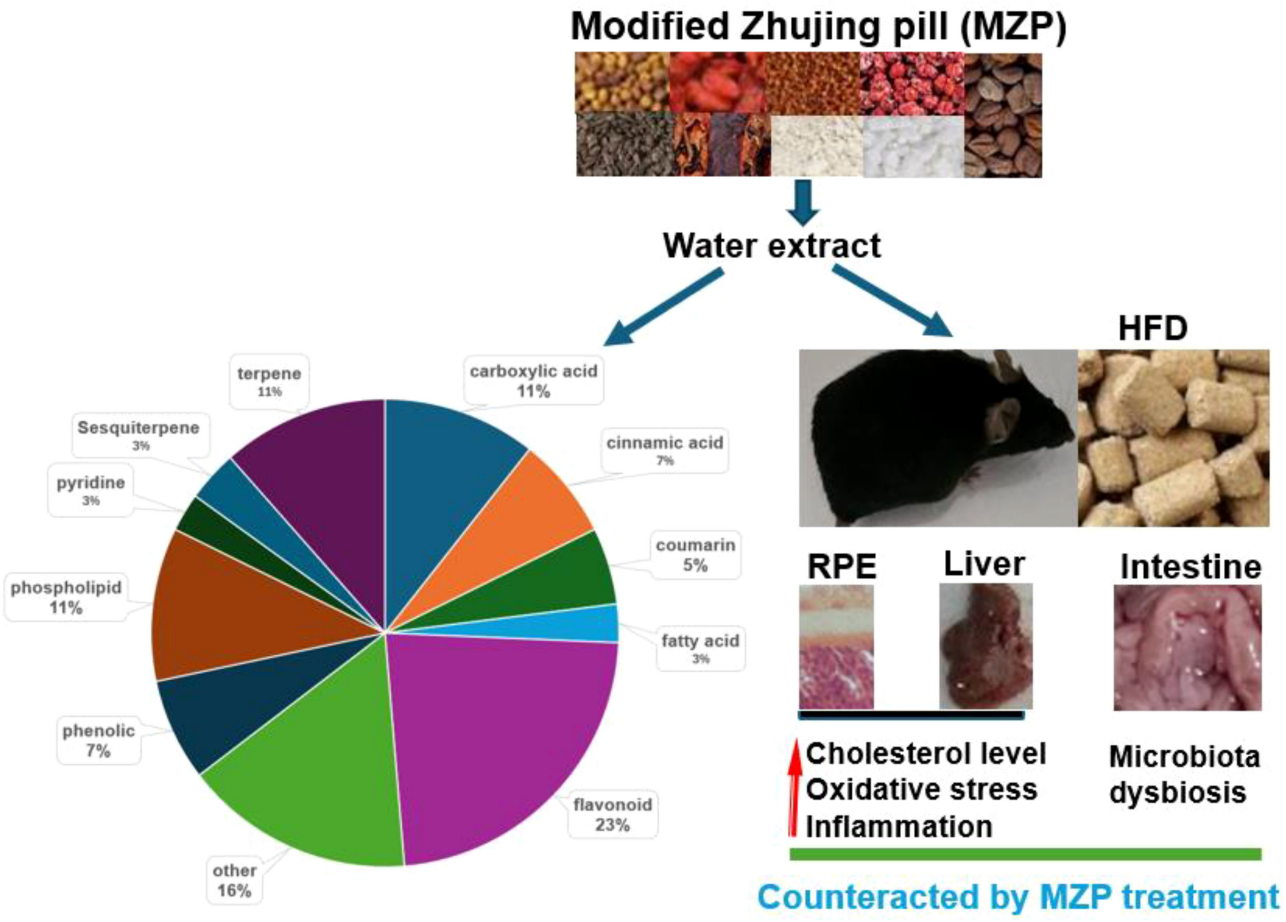
Protective mechanisms of MZP against AMD. HFD can induce cholesterol accumulation, oxidative stress, inflammation, and gut microbiota dysbiosis. MZP can counteract HFD-induced toxic effects mainly via polyphenols. AMD, age-related macular degeneration; HFD, high-fat diet; MZP, modified Zhujing pill; RPE, retinal pigment epithelial cells.

The purpose of this study was to unravel the protective mechanisms of MZP against AMD in HFD-fed animals. This study demonstrated that HFD increased the cholesterol level in the RPE, liver, and serum and was associated with oxidative stress and inflammation. HFD also caused gut microbiota dysbiosis. MZP treatment reversed the HFD-induced effects. It is possible that dysregulation of cholesterol metabolism and elevated oxidative stress and inflammation are associated with abnormal changes in gut bacterial composition and metabolic pathways. Polyphenols, as predominantly identified compounds in MZP, are suggested to play a critical role in counteracting HFD-induced toxic effects via regulation of cholesterol metabolism, inhibition of oxidative stress and inflammation, and modulation of gut microbiota (**Figure 11**).

## Data Availability

The data presented in this study are deposited in the NCBI Bioproject database with Accession Number PRJNA1347468.
