# Supplementary material for: Modified Zhujing pill regulates RPE cholesterol metabolism and gut microbiota in an age-related macular degeneration mouse model
Source: Front Cell Infect Microbiol. 2025 Oct 31;15:1691360. doi: 10.3389/fcimb.2025.1691360 (PMC12615425; doi:10.3389/fcimb.2025.1691360)
Supplement: Supplementary file 1 [file Table1.docx]

**Supplementary data**

**
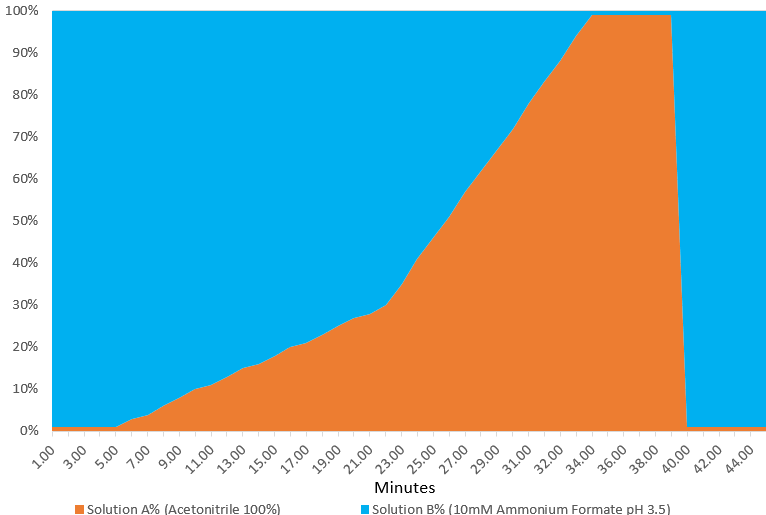
**

**Figure S1** Elution Program showing the changes in percentage of Solution A and B during the HPLC running process.


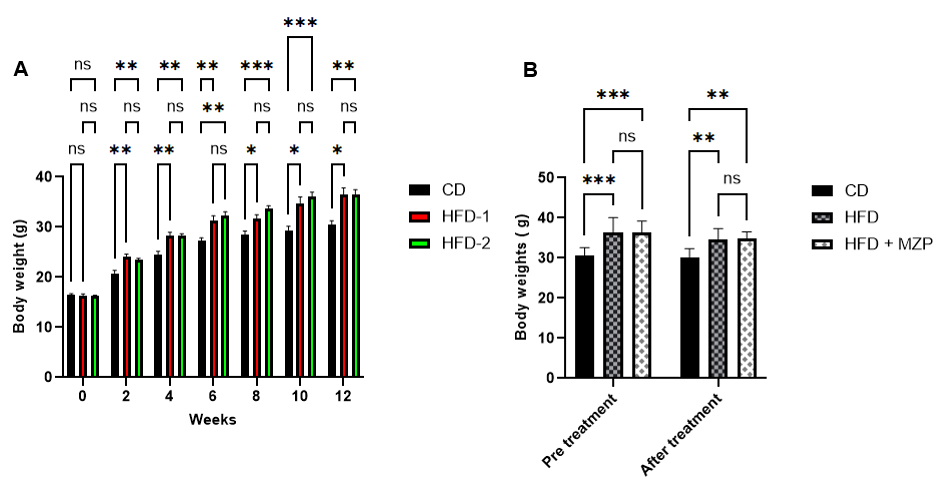


**Figure S2** Mouse body weight was monitored every two weeks and analysed by two-way ANOVA followed by a Bonferroni test. (A) Body weight of three groups when feeding control diet (CD) or high-fat diet (HFD). (B) HFD-2 received treatment with MZP, HFD-1 and CD groups received vehicle treatment for four weeks. Body weight of three groups’ animas was measured pre-treatment and after-treatment. **p*<0.05, ** *p*<0.01, *** *p*<0.001; ns: no significance. MZP, modified Zhujing pill.

**
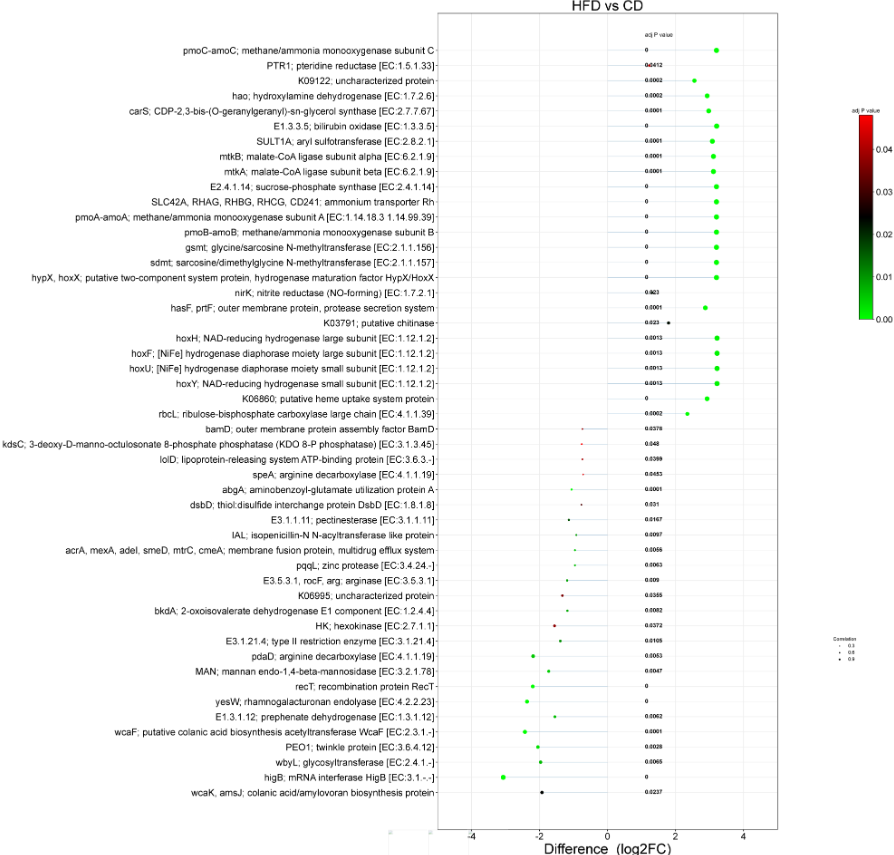
**

**
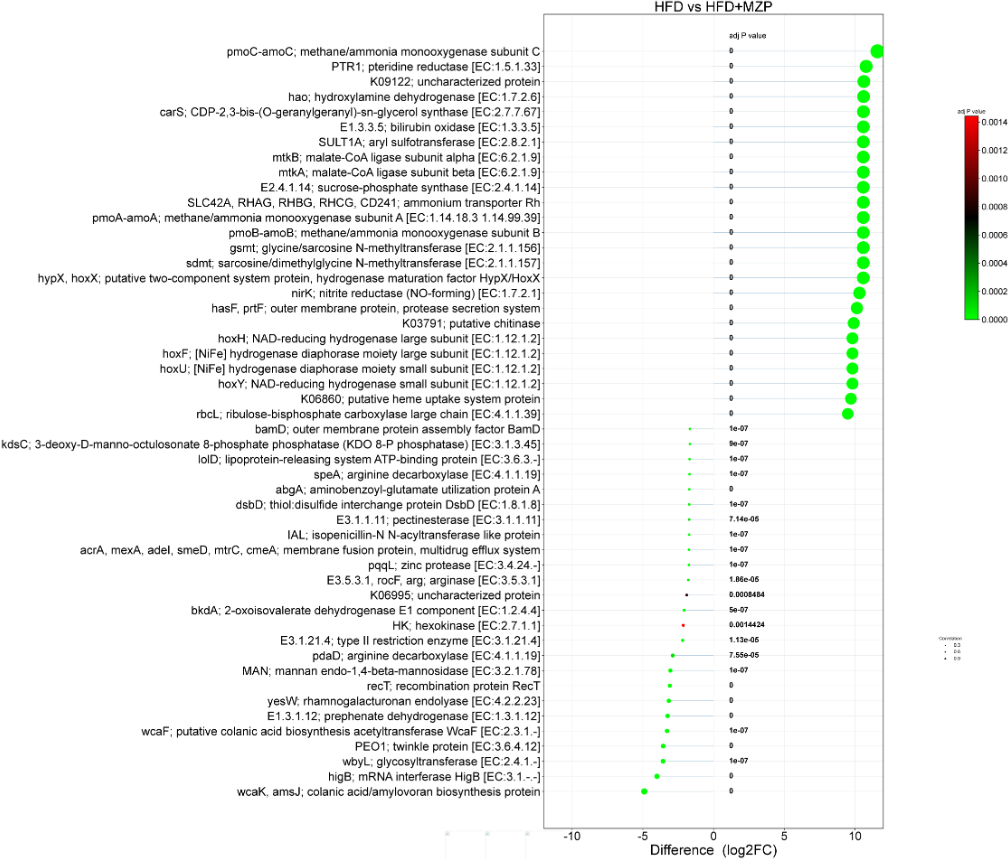
**

**
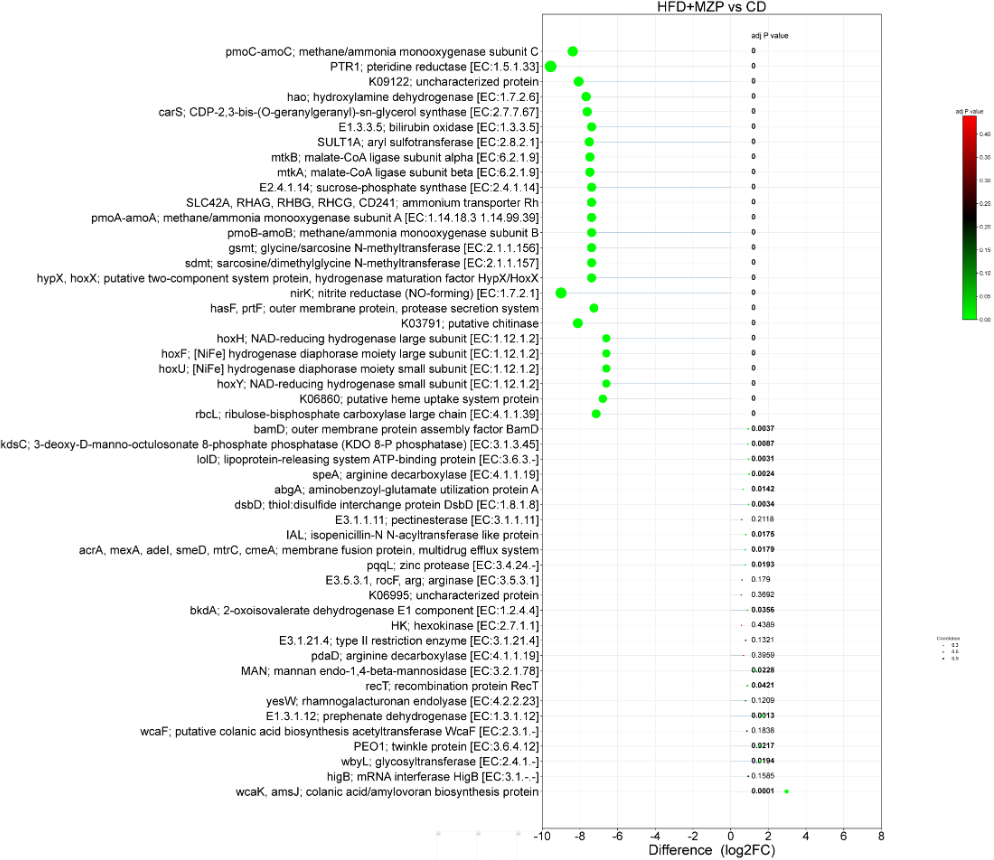
**

**Figure S3** Comparison of top 25 upregulated and 25 downregulated KOs between individual experimental groups.

**Table S1** Sequences of primers used in qRT-PCR

| Gene | Forward Primer (5’-3’) | Reverse Primer (5’-3’) |
| --- | --- | --- |
| *Abca1* | AGTTTCGGTATGGCGGGTTT | AGCATGCCAGCCCTTGTTAT |
| *Abcg1* | ACCTACCACAACCCAGCAGACTTT | GGTGCCAAAGAAACGGGTTCACAT |
| *Cyp27a1* | GCCTTGCACAAGGAAGTGACT | CGCAGGGTCTCCTTAATCACA |
| *Cyp46a1* | CCCTAGCCTTTCCCCAAATTGC | CGGAAGAATCCCTTGCAACC |
| *Nr1h3*  *(coding* LXRα*)* | CCCTAGCCTTTCCCCAAATTGC | CGGAAGAATCCCTTGCAACC |
| *Catalase* | GCGGACATTCTACACAAAGGT | CAGTGAAATTCTTGACCGCTT |
| *Gpx1* | CGGAATGCCTTGCCAACACC | CAAAGTTCCAGGCAATGTCGTT |
| *Sod1* | GAACCATCCACTTCGAGCAG | CAACATGCCTCTCTTCATCCG |
| *Il-1 β* | CAGGCAGGCAGTATCACTCA | AGCTCATATGGGTCCGACAG |
| *Il-6* | TACCACTTCACAAGTCG | CTGCAAGTGCATCATCG |
| *Tnf-α* | ACGTGGAACTGGCAGAAGAG | AGGGTCTGGGCCATAGAACT |
| *Gapdh* | CCCACTAACATCAAATGGGG | CCTTCCACAATGCCAAAGTT |

**Table S2** HPLC-MS/MS positive ion mode analysis identified compounds based on area under curve

| m/z | RT | Area | Height | Spectral match |
| --- | --- | --- | --- | --- |
| 233.1535 | 26.77 | 2.50E+06 | 3.50E+07 | (+)-Costunolide |
| 251.2024 | 27.77 | 4.20E+05 | 3.10E+06 | (3aR)-(+)-Sclareolide |
| 279.2302 | 32.96 | 7.90E+06 | 2.40E+07 | ?-linolenic acid |
| 760.5828 | 31.82 | 3.40E+07 | 1.10E+08 | 1-(9Z-octadecenoyl)-2-hexadecanoyl-sn-glycero-3-phosphocholine |
| 762.5899 | 31.72 | 4.20E+06 | 1.40E+07 | 1,2-diheptadecanoyl-sn-glycero-3-phosphocholine |
| 786.5988 | 32.38 | 1.90E+07 | 6.20E+07 | 1,2-Dioleoyl-sn-glycero-3-phosphocholine |
| 510.3555 | 28.36 | 3.90E+06 | 4.30E+07 | 1-heptadecanoyl-2-hydroxy-sn-glycero-3-phosphocholine |
| 782.5651 | 33.44 | 2.90E+05 | 3.00E+06 | 1-hexadecanoyl-2--(5Z,8Z,11Z,14Z-eicosatetraenoyl)-sn-glycero-3-phosphocholine |
| 760.574 | 29.35 | 1.90E+07 | 7.30E+07 | 1-hexadecanoyl-2-(9Z-octadecenoyl)-sn-glycero-3-phosphocholine |
| 496.3394 | 28.06 | 1.40E+08 | 1.40E+09 | 1-Hexadecanoyl-sn-glycero-3-phosphocholine |
| 468.3095 | 27.51 | 1.50E+06 | 2.10E+07 | 1-Myristoyl-sn-glycero-3-phosphocholine |
| 546.3521 | 28.76 | 9.80E+06 | 7.30E+07 | 1-octadecanoyl-sn-glycero-3-phosphocholine |
| 524.3609 | 28.19 | 5.80E+06 | 5.70E+07 | 1-Stearoyl-sn-glycero-3-phosphocholine |
| 158.0963 | 13.64 | 5.80E+05 | 1.70E+06 | 2,6-Dimethylquinoline |
| 146.0921 | 4.24 | 3.00E+06 | 2.20E+07 | 4-guanidinobutanoic acid |
| 202.1798 | 6.87 | 3.30E+05 | 5.80E+06 | 4-Hydroxy-1-(2-hydroxyethyl)-2,2,6,6-tetramethylpiperidine |
| 179.0696 | 21.74 | 5.40E+05 | 2.30E+06 | 4-Hydroxy-3-methoxycinnamaldehyde |
| 177.0542 | 8.7 | 3.10E+06 | 1.70E+07 | 4-Methylumbelliferone |
| 156.0807 | 24.45 | 2.20E+05 | 2.50E+06 | 4-Phenylpyridine |
| 207.0651 | 24.91 | 4.50E+05 | 6.10E+06 | 5,7-Dimethoxy-2H-chromen-2-one |
| 237.1846 | 26.91 | 4.00E+06 | 4.10E+07 | 5-hydroxyculmorin |
| 433.1119 | 23.51 | 3.60E+05 | 3.70E+06 | Apigenin-7-O-glucoside |
| 355.1016 | 9.72 | 8.20E+06 | 3.20E+07 | Caffeoyl quinic acid |
| 193.0341 | 3.16 | 5.70E+06 | 2.70E+07 | Citric acid |
| 179.0335 | 12.01 | 5.00E+05 | 1.80E+06 | Daphnetin |
| 231.138 | 27.21 | 3.10E+06 | 4.80E+07 | Dehydrocostus lactone |
| 130.0862 | 3.18 | 3.80E+06 | 2.80E+07 | DL-Pipecolinic acid |
| 183.086 | 1.95 | 2.50E+07 | 3.20E+08 | D-Sorbitol |
| 307.2623 | 29.83 | 3.30E+05 | 3.20E+06 | Eicosatrienoic acid |
| 307.1756 | 12.53 | 9.00E+05 | 4.50E+06 | Feruloyl agmatine (isomer of 1608) |
| 323.1705 | 10.85 | 1.90E+06 | 8.20E+06 | Feruloyl lysine |
| 265.1547 | 10.3 | 1.20E+06 | 8.80E+06 | Feruloyl putrescine (isomer of 1173) |
| 314.1364 | 23.79 | 1.70E+06 | 1.90E+07 | Feruloyl tyramine |
| 357.1167 | 8.7 | 5.50E+05 | 3.30E+06 | Gentiopicroside |
| 471.345 | 28.28 | 1.30E+06 | 1.10E+07 | Glycyrrhetinic Acid |
| 465.1024 | 22.32 | 9.20E+05 | 5.30E+06 | Hyperoside |
| 176.0703 | 22.41 | 2.70E+05 | 1.60E+06 | Indolacetic acid |
| 146.0603 | 21.05 | 1.90E+06 | 5.20E+06 | Indole-3-carboxyaldehyde |
| 317.065 | 25.31 | 6.60E+05 | 7.60E+06 | Isorhamnetin |
| 479.1182 | 23.79 | 1.30E+06 | 1.20E+07 | Isorhamnetin-3-O-glucoside |
| 287.0544 | 25.2 | 2.60E+06 | 2.80E+07 | Kaempferol |
| 449.1075 | 23.77 | 1.40E+06 | 8.50E+06 | Kaempferol-3-O-glucoside |
| 281.2466 | 29.04 | 1.10E+06 | 7.60E+06 | Linoleic acid |
| 243.0872 | 23.96 | 7.20E+05 | 8.00E+06 | Lumichrome |
| 123.0554 | 7.43 | 4.50E+06 | 2.10E+07 | Niacinamide |
| 124.0393 | 4.42 | 2.60E+06 | 8.80E+06 | Nicotinic acid |
| 219.1741 | 27.75 | 1.10E+08 | 9.40E+08 | Nootkatone |
| 184.0731 | 1.84 | 2.80E+06 | 6.60E+07 | O-Phosphocholine |
| 245.1163 | 26.68 | 7.20E+05 | 1.20E+07 | Osthole |
| 220.1176 | 8.62 | 3.00E+06 | 1.50E+07 | Pantothenate |
| 597.1437 | 22.32 | 4.80E+06 | 2.80E+07 | Peltatoside |
| 303.0491 | 23.08 | 3.20E+06 | 2.20E+07 | Quercetin |
| 627.1535 | 10.88 | 5.80E+05 | 2.60E+06 | Quercetin-3,4'-O-di-beta-glucoside |
| 377.1446 | 14.98 | 1.40E+05 | 7.60E+05 | Riboflavin |
| 611.1589 | 23.12 | 5.00E+05 | 4.10E+06 | Rutine |
| 139.0391 | 2.04 | 3.10E+05 | 4.70E+06 | Salicylic acid |
| 433.2218 | 33.81 | 2.40E+06 | 1.90E+07 | Schizandrin |
| 193.0493 | 17.79 | 6.20E+06 | 2.00E+07 | Scopoletin |
| 258.111 | 2.49 | 1.20E+06 | 3.40E+06 | sn-Glycero-3-phosphocholine |
| 231.1124 | 11.44 | 2.00E+05 | 8.40E+05 | Tetrahydroharman-3-carboxylic acid |
| 138.0548 | 2.12 | 2.90E+07 | 1.70E+08 | Trigonelline |
| 253.1797 | 25.66 | 2.00E+06 | 5.00E+06 | Walleminone |
| 285.075 | 26.13 | 1.20E+06 | 1.60E+07 | Wogonin |

**Table S3** HPLC-MS/MS negative ion mode analysis identified compounds based on area under curve

| m/z | RT | Area | Height | Ion identity | Spectral match |
| --- | --- | --- | --- | --- | --- |
| 147.0286 | 4.62 | 4.90E+06 | 2.60E+07 |  | (-)-Citramalic acid |
| 564.3318 | 27.77 | 2.20E+06 | 2.90E+07 |  | 1-(9Z,12Z-Octadecadienoyl-2-hydroxy-sn-glycero-3-phosphocholine |
| 478.2943 | 28.2 | 1.90E+05 | 2.20E+06 |  | 1-(9Z-octadecenoyl)-sn-glycero-3-phosphoethanolamine |
| 540.3331 | 27.99 | 9.80E+05 | 1.10E+07 |  | 1-Hexadecanoyl-sn-glycero-3-phosphocholine |
| 175.0602 | 10 | 1.30E+07 | 3.70E+07 |  | 2-Isopropylmalic acid |
| 179.0339 | 12.57 | 3.50E+06 | 1.40E+07 |  | 3,4-dihydroxycinnamic acid |
| 263.1299 | 23.6 | 9.90E+04 | 8.80E+05 |  | Abscisic acid |
| 283.061 | 26.3 | 4.70E+05 | 7.00E+06 |  | Acacetin |
| 134.0458 | 6.45 | 1.80E+06 | 6.60E+06 | [M-H]- | Adenine |
| 269.0458 | 25.46 | 1.20E+06 | 8.60E+06 |  | Apigenin |
| 132.0288 | 1.83 | 3.50E+05 | 6.50E+06 |  | Aspartate |
| 315.0721 | 7.91 | 1.20E+05 | 9.00E+05 |  | Benzoic acid + 2O, O-Hex |
| 341.0875 | 9.26 | 2.20E+05 | 1.50E+06 |  | Caffeic acid hexoside |
| 353.0879 | 8.64 | 1.40E+06 | 7.90E+06 |  | Caffeoyl quinic acid (isomer of 832, 833, 834) |
| 339.1999 | 31.44 | 1.60E+06 | 1.90E+06 |  | Canrenone |
| 253.0518 | 26.28 | 2.20E+05 | 2.60E+06 |  | Chrysin |
| 191.019 | 8.71 | 5.30E+05 | 3.50E+06 |  | Citric acid |
| 337.0931 | 11.94 | 6.50E+05 | 2.30E+06 |  | Coumaroyl quinic acid (isomer of 758, 760) |
| 193.0347 | 1.9 | 9.30E+05 | 8.60E+06 |  | D(+)-Galacturonic acid |
| 133.0128 | 2.18 | 5.80E+07 | 7.00E+08 |  | D-(+)-Malic acid |
| 503.1623 | 2.1 | 4.70E+05 | 9.10E+06 | [M-H]- | D-(+)-Raffinose |
| 253.0513 | 24.32 | 1.40E+05 | 1.40E+06 |  | Daidzein |
| 201.1125 | 24.78 | 1.60E+06 | 1.60E+07 |  | Decanedioic acid |
| 195.0501 | 2.09 | 1.30E+07 | 1.00E+08 |  | D-Gluconic acid |
| 515.1197 | 22.14 | 4.90E+04 | 3.50E+05 |  | Dicaffeoyl quinic acid |
| 229.1441 | 25.91 | 2.70E+05 | 2.50E+06 |  | Dodecanedioic acid |
| 218.1031 | 8.63 | 6.00E+05 | 2.80E+06 |  | D-Pantothenic Acid |
| 181.0707 | 1.95 | 4.40E+07 | 7.00E+08 | [M-H]- | D-Sorbitol |
| 449.1098 | 23.45 | 2.90E+05 | 2.40E+06 |  | Eriodictyol-7-O-glucoside |
| 177.0183 | 12.01 | 2.10E+06 | 8.40E+06 |  | Esculetin |
| 355.1032 | 11.22 | 1.30E+05 | 3.60E+05 |  | Feruloyl Hexoside (isomer of 847) |
| 367.1035 | 12.72 | 1.20E+05 | 3.60E+05 |  | Feruloyl quinic acid (isomer of 886, 888) |
| 267.0672 | 25.62 | 1.10E+05 | 1.30E+06 |  | Formononetin |
| 169.0131 | 7.59 | 1.10E+06 | 8.10E+06 |  | Gallic acid |
| 783.4893 | 27.5 | 1.60E+06 | 1.20E+07 |  | Ginsenoside F2 |
| 991.5511 | 26.74 | 1.10E+06 | 1.30E+07 |  | Ginsenoside Re |
| 845.4947 | 25.96 | 1.20E+05 | 1.70E+06 |  | Ginsenoside Rf |
| 829.4985 | 25.85 | 2.50E+06 | 3.30E+07 |  | Ginsenoside Rg2 |
| 829.497 | 27.51 | 3.10E+07 | 2.30E+08 |  | Ginsenoside Rg3(R-FORM) |
| 811.4867 | 27.87 | 1.40E+07 | 1.10E+08 |  | Ginsenoside Rg5 |
| 811.4878 | 26.73 | 2.60E+06 | 1.90E+07 | [M+FA]- | Ginsenoside Rg6 |
| 845.4922 | 24.61 | 1.50E+06 | 1.50E+07 |  | Ginsenoside-Rg1 |
| 559.3125 | 27.61 | 2.00E+06 | 2.20E+07 |  | Glc-octadecatrienoyl-sn-glycerol (isomer 1) (PUT) |
| 282.0844 | 7.67 | 1.60E+05 | 1.90E+06 |  | Guanosine |
| 344.0397 | 7.24 | 5.70E+04 | 9.80E+05 |  | Guanosine-3',5'-cyclic monophosphate |
| 189.0759 | 14.67 | 9.90E+06 | 2.60E+07 |  | Hydroxysuberic acid |
| 255.0667 | 25.29 | 1.30E+05 | 1.60E+06 |  | isoliquiritigenin |
| 315.0512 | 25.32 | 1.30E+06 | 1.40E+07 |  | Isorhamnetin |
| 477.1046 | 23.79 | 9.90E+05 | 8.40E+06 |  | Isorhamnetin-3-O-glucoside |
| 285.0404 | 25.21 | 4.40E+06 | 4.60E+07 |  | Kaempferol |
| 447.0943 | 23.78 | 1.10E+06 | 6.10E+06 |  | Kaempferol-3-O-glucoside |
| 821.3976 | 26.05 | 2.60E+05 | 3.20E+06 |  | Licoricesaponin H2 |
| 417.1203 | 21.55 | 2.70E+05 | 9.50E+05 | [M-H]- | Liquiritin |
| 285.0404 | 24.84 | 5.30E+05 | 5.50E+06 |  | Luteolin |
| 343.1237 | 2.12 | 3.10E+05 | 5.30E+06 | [M-H]- | Maltitol |
| 209.0296 | 1.84 | 9.10E+05 | 1.70E+07 |  | Mucic acid |
| 188.0556 | 4.6 | 4.70E+05 | 2.40E+06 |  | N-Acetyl-DL-glutamic acid |
| 271.0611 | 24.64 | 2.10E+05 | 2.20E+06 |  | Naringenin |
| 292.1401 | 7.17 | 1.30E+06 | 3.60E+06 |  | N-Fructosyl isoleucine |
| 290.0884 | 2.18 | 2.90E+06 | 4.00E+07 |  | N-Fructosyl pyroglutamate |
| 341.1085 | 2.3 | 1.20E+06 | 6.10E+06 | [M-H]- | Palatinose |
| 163.0389 | 17.9 | 5.80E+06 | 1.70E+07 |  | p-Coumaric acid |
| 595.1308 | 22.33 | 6.60E+06 | 3.00E+07 | [M-H]- | Peltatoside |
| 301.0347 | 24.58 | 9.70E+06 | 9.00E+07 |  | Quercetin |
| 609.146 | 23.12 | 6.20E+05 | 4.60E+06 |  | Quercetin 3-O-[2''-O-b-D-glucopyranosyl]-a-L-rhamnopyranoside |
| 625.1412 | 10.87 | 2.80E+05 | 1.20E+06 | [M-H]- | Quercetin-3,4'-O-di-beta-glucoside |
| 433.0771 | 23.43 | 5.50E+04 | 3.90E+05 |  | Quercetin-3-Arabinoside |
| 463.089 | 23.08 | 1.90E+07 | 1.20E+08 | [M-H]- | Quercetin-3-O-beta-D-galactoside |
| 505.0997 | 23.66 | 5.00E+04 | 5.00E+05 |  | Quercetin-3-O-glucose-6''-acetate |
| 315.0512 | 25.62 | 7.50E+04 | 9.70E+05 |  | Rhamnetin |
| 359.0772 | 22.57 | 9.70E+04 | 7.20E+05 |  | Rosmarinic acid |
| 173.0444 | 2.88 | 7.30E+06 | 4.50E+07 |  | Shikimic acid |
| 463.0886 | 24 | 3.80E+04 | 3.80E+05 |  | Spiraeoside |
| 173.0809 | 21.53 | 1.80E+06 | 5.60E+06 |  | Suberic acid |
| 117.0179 | 2.25 | 3.60E+06 | 2.10E+07 |  | Succinic acid |
| 341.1083 | 2.67 | 2.80E+06 | 2.40E+07 | [M-H]- | Sucrose |
| 593.1291 | 24.54 | 2.80E+05 | 2.50E+06 |  | Tiliroside |
| 215.1283 | 25.42 | 6.50E+05 | 6.20E+06 |  | Undecanedioic acid |
| 243.0626 | 7 | 6.60E+05 | 9.60E+06 | [M-H]- | Uridine |
| 323.028 | 5.38 | 4.60E+04 | 3.10E+05 |  | Uridine 5'-monophosphate |
| 283.0609 | 26.12 | 9.60E+05 | 1.20E+07 |  | Wogonin |
